# eIF2B-capturing viral protein NSs suppresses the integrated stress response

**DOI:** 10.1038/s41467-021-27337-x

**Published:** 2021-12-07

**Authors:** Kazuhiro Kashiwagi, Yuichi Shichino, Tatsuya Osaki, Ayako Sakamoto, Madoka Nishimoto, Mari Takahashi, Mari Mito, Friedemann Weber, Yoshiho Ikeuchi, Shintaro Iwasaki, Takuhiro Ito

**Affiliations:** 1 Laboratory for Translation Structural Biology, RIKEN Center for Biosystems Dynamics Research, Tsurumi-ku, Yokohama, 230-0045 Japan; 2 RNA Systems Biochemistry Laboratory, RIKEN Cluster for Pioneering Research, Wako, Saitama 351-0198 Japan; 3 Institute of Industrial Science, The University of Tokyo, Meguro-ku, Tokyo, 153-8505 Japan; 4 Institute for Virology, FB10-Veterinary Medicine, Justus-Liebig University, Giessen, D-35392 Germany; 5 Institute for AI and Beyond, The University of Tokyo, Bunkyo-ku, Tokyo, 113-8655 Japan; 6 Department of Computational Biology and Medical Sciences, Graduate School of Frontier Sciences, The University of Tokyo, Kashiwa, Chiba, 277-8561 Japan

**Keywords:** Translation, Cryoelectron microscopy

## Abstract

Various stressors such as viral infection lead to the suppression of cap-dependent translation and the activation of the integrated stress response (ISR), since the stress-induced phosphorylated eukaryotic translation initiation factor 2 [eIF2(αP)] tightly binds to eIF2B to prevent it from exchanging guanine nucleotide molecules on its substrate, unphosphorylated eIF2. Sandfly fever Sicilian virus (SFSV) evades this cap-dependent translation suppression through the interaction between its nonstructural protein NSs and host eIF2B. However, its precise mechanism has remained unclear. Here, our cryo-electron microscopy (cryo-EM) analysis reveals that SFSV NSs binds to the α-subunit of eIF2B in a competitive manner with eIF2(αP). Together with SFSV NSs, eIF2B retains nucleotide exchange activity even in the presence of eIF2(αP), in line with the cryo-EM structures of the eIF2B•SFSV NSs•unphosphorylated eIF2 complex. A genome-wide ribosome profiling analysis clarified that SFSV NSs expressed in cultured human cells attenuates the ISR triggered by thapsigargin, an endoplasmic reticulum stress inducer. Furthermore, SFSV NSs introduced in rat hippocampal neurons and human induced-pluripotent stem (iPS) cell-derived motor neurons exhibits neuroprotective effects against the ISR-inducing stress. Since ISR inhibition is beneficial in various neurological disease models, SFSV NSs may be a promising therapeutic ISR inhibitor.

## Abstract (web summary)

Here the authors show that a viral protein interferes with the binding of phosphorylated eIF2 to eIF2B, thereby suppressing the host integrated stress response (ISR). This suppression of the ISR abrogates translational changes of the host and ameliorates neurite degradation under stress.

## Introduction

Upon various stresses, eukaryotic cells activate a common pathway called the integrated stress response (ISR), a homeostatic pathway necessary for organismal fitness. In vertebrates, four protein kinases, EIF2AK1 to EIF2AK4 (also known as HRI, PKR, PERK, and GCN2), which are activated by different stress stimuli, commonly phosphorylate the α-subunit of eukaryotic initiation factor 2 (eIF2). Diverse stress signals are thus integrated into the phosphorylation of eIF2 and activate the common downstream pathway^1^.

eIF2 is a heterotrimeric G-protein that delivers an initiator methionyl-transfer RNA (Met-tRNA_i_) to 40S ribosomes in a GTP-dependent manner and is also involved in the start codon recognition process on the ribosomes. After the recognition, eIF2 is released from the ribosomes as the guanosine disphosphate (GDP)-bound form and regenerated by its specific guanine nucleotide exchange factor, eIF2B, a heterodecameric complex of two copies each of the α-, β-, γ-, δ-, and ε-subunits^2^. However, phosphorylated eIF2 [eIF2(αP)] binds to eIF2B in an alternative nonproductive mode and interferes with the guanine nucleotide exchange reaction of eIF2B^3,4^. As eIF2B is less abundant than eIF2 in cells, the phosphorylation of a portion of eIF2 can sufficiently inhibit the catalytic activity of eIF2B^2^. This inhibition limits the supply of active GTP-bound eIF2, resulting in the global attenuation of protein synthesis and the selective translation of stress-related mRNAs^5^.

Infection by various DNA and RNA viruses is one of the drivers of the ISR program. PKR is activated by double-stranded RNA derived from the virus, and resultant phosphorylates eIF2 shuts off global protein synthesis^6^. On the other hand, viruses have also evolved several strategies to evade this host defense mechanism. For example, poliovirus induces the degradation of PKR^7^, vaccinia virus blocks the phosphorylation of eIF2 by producing a pseudo-substrate for PKR^8,9^, and herpes simplex virus induces the dephosphorylation of eIF2(αP)^10^. Recently, nonstructural protein S(NSs) of sandfly fever Sicilian virus (SFSV) was shown to employ a novel mechanism to evade the protein synthesis arrest^11^. SFSV belongs to the genus *Phlebovirus* (order *Bunyavirales*) and is endemic in the Mediterranean region^12^. During the SFSV infection, PKR activation and eIF2 phosphorylation are both induced. However, the viral NSs protein counteracts the attenuation of cap-dependent translation. As SFSV NSs binds to eIF2B, it may force eIF2B into the productive mode^11^. Proteins from beluga whale coronavirus and Aichi picornavirus also reportedly target eIF2B^13^. However, their molecular mechanisms have not been elucidated in detail.

The properties of SFSV NSs, which directly binds to eIF2B and prevents the attenuation of cap-dependent translation, are somewhat similar to those of ISRIB, a small molecule that acts as an allosteric eIF2(αP) antagonist. ISRIB-bound eIF2B disfavors the interaction with eIF2(αP)^14,15^. Interestingly, ISRIB has shown promising effects in various animal models of neuropathological conditions, such as traumatic brain injury^16^, amyotrophic lateral sclerosis^17^, Down syndrome^18^, and prion disease^19^, as well as in the normal aging process^20^. The resemblance of SFSV NSs and ISRIB led us to investigate how SFSV NSs binds to eIF2B to antagonize eIF2(αP) and whether SFSV NSs attenuates the ISR.

Here we determined the cryo-electron microscopy (cryo-EM) structure of the eIF2B•SFSV NSs complex, which revealed that SFSV NSs sterically blocks eIF2(αP) binding to eIF2B. Biochemical experiments showed that SFSV NSs almost nullifies the inhibitory effect of eIF2(αP) without affecting the guanine nucleotide exchange on eIF2 catalyzed by eIF2B, which was further exemplified by the cryo-EM structure of the eIF2B•SFSV NSs•unphosphorylated eIF2 complex. A ribosome profiling analysis revealed that SFSV NSs expressed in cultured human cells attenuates the ISR effectively. Furthermore, SFSV NSs expressed in rat hippocampal neurons or human iPS cell-derived motor neurons exhibited neuroprotective effects against the ISR-inducing stress, which indicates a potential of SFSV NSs as a therapeutic ISR inhibitor.

## Results and discussion

### SFSV NSs and eIF2(αP) bind to overlapping regions on eIF2B

**Fig. 1:**
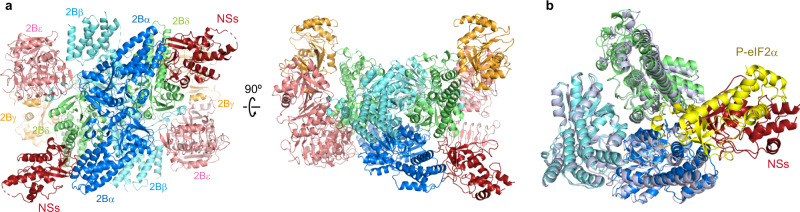
Cryo-EM structure of the human eIF2B SFSV NSs complex. **a** Overall structure of the human eIF2B•SFSV NSs complex (eIF2Bα: blue; eIF2Bβ: cyan; eIF2Bγ: orange; eIF2Bδ: lime; eIF2Bε: pink; SFSV NSs: maroon). **b** Overlay showing a comparison of the interactions through the α-δ groove of eIF2B in the eIF2B•SFSV NSs complex and in the eIF2B•eIF2(αP) complex (PDB: 7D44)^14^. The eIF2B subunits and the phosphorylated eIF2α subunit in the eIF2B•eIF2(αP) complex are pale blue and yellow, respectively. The two structures are aligned with their eIF2Bβ subunit C-terminal domains.

We successfully purified the SFSV NSs protein (Supplementary Fig. 1a) and determined the complex structure of human eIF2B and SFSV NSs by cryo-EM at 2.3 Å resolution (Fig. 1, Supplementary Fig. 1, and Supplementary Table 1). Two SFSV NSs molecules bind one eIF2B molecule and are located between the α- and δ-subunits of eIF2B (Fig. 1a). The interfaces for SFSV NSs partially overlap with those of the phosphorylated eIF2α subunit in the eIF2B•eIF2(αP) complex^14^, but there are no similarities between phosphorylated eIF2α and SFSV NSs in terms of their three-dimensional structures and interaction modes with eIF2B (Fig. 1b).

We were able to assign the amino (N)-terminal ~200 residues out of the 261 residues of SFSV NSs in the cryo-EM map (Supplementary Fig. 1h). The N-terminal region of SFSV NSs contains a β-sheet, followed by a helical core. The arrangement of the helices is similar to that in the core domain of the NSs protein from the Rift Valley fever virus^21^, another virus in the genus *Phlebovirus*, which targets PKR^22,23^ (Supplementary Fig. 1i). The interaction of SFSV NSs with eIF2B is mediated by the N-terminal β-sheet region. The β-sheets and protruding loops form a concave interface that embraces helix α3 in the N-terminal domain of the eIF2Bα subunit. The very N terminus of SFSV NSs is also involved in the interaction with eIF2Bα (Supplementary Fig. 1j). Therefore, if a peptide artificially extends the N terminus, it would interfere with the binding to eIF2Bα. This explains the previous observation that the N-terminally tagged version of the SFSV NSs protein completely lost its activity and ability to interact with eIF2B^11^. The eIF2B interaction with SFSV NSs is mostly mediated by the α-subunit and does not induce a large movement of the eIF2B subunits, as compared with the apo eIF2B structure^14^ (Supplementary Fig. 1k). This is in stark contrast to phosphorylated eIF2α, which binds like a wedge between the α- and δ-subunits of eIF2B and interacts with both subunits extensively, resulting in the structural rearrangement of the eIF2B subunits^14,15^ (Fig. 1b and Supplementary Fig. 1k).

**Fig. 2:**
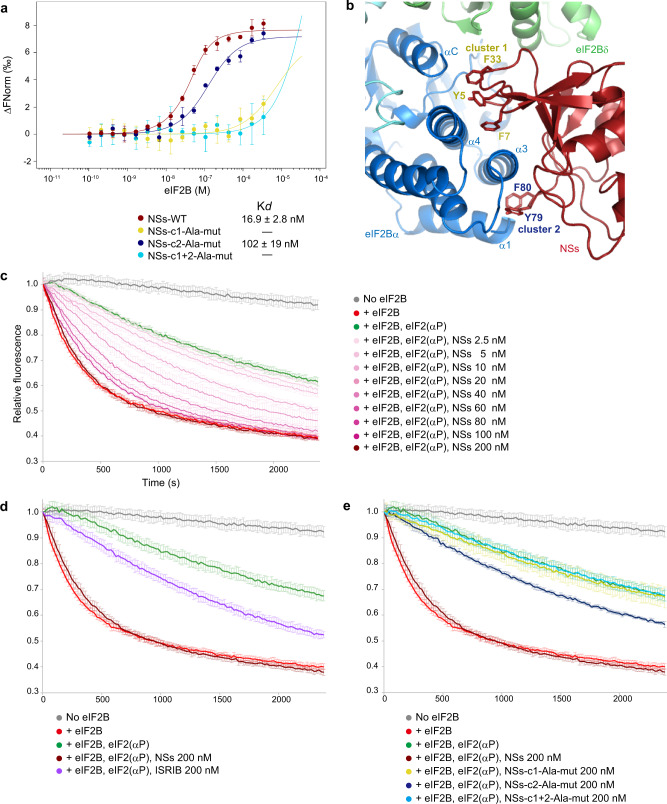
Aromatic clusters of SFSV NSs are important for binding to eIF2B and suppressing the inhibitory effect of eIF2(αP). **a** MST analysis between eIF2B and SFSV NSs. Fluorescently labeled SFSV NSs (100 nM) was mixed with an equal volume of a 16-step serial dilution of 6.8 μM eIF2B and the microscale thermophoresis was measured. Plots of the wild-type SFSV NSs (maroon) and the alanine-substituted mutants on aromatic cluster 1 (SFSV NSs-c1-Ala-mut: Y5A, F7A, and F33A; yellow), aromatic cluster 2 (SFSV NSs-c2-Ala-mut: Y79A and F80A; blue), and both clusters (SFSV NSs-c1 + 2-Ala-mut: Y5A, F7A, F33A, Y79A, and F80A; cyan) are shown. Data are presented as mean values ± SDs at each concentration, and *n* = 3 independent experiments. **b** Two aromatic clusters of SFSV NSs at the interface with eIF2B. Aromatic cluster 1 (c1: Y5, F7, and F33) and aromatic cluster 2 (c2: Y79 and F80) grasp the α3 helix of the eIF2Bα subunit from both sides and bury the space between the helices of the eIF2Bα subunit. **c**–**e** Guanine nucleotide exchange assay. Non-phosphorylatable eIF2(αS51A) (final 150 nM) was loaded with BODIPY-GDP and fluorescent signals were read every 20 s. In the three panels, the gray lines are the measurements without eIF2B, and other measurements were started by the addition of eIF2B (final 40 nM). The red and green lines are the measurements without and with eIF2(αP) (final 1.5 μM), respectively. In the experiments shown in **c**, various concentrations (0–200 nM) of wild-type SFSV NSs were included in the reaction solutions containing fluorescent-eIF2(αS51A) and eIF2(αP). In the experiments shown in (**d** and **e**), ISRIB (**d**) or the mutants of SFSV NSs (c1-Ala-mut, c2-Ala-mut, and c1 + 2-Ala-mut) (**e**) were included into the reaction solutions at 200 nM. The same controls (No eIF2B, eIF2B, eIF2B + eIF2(αP) and eIF2B + eIF2(αP) + SFSV NSs at 200 nM) were used in (**d** and **e**). Data are presented as mean values ± SDs at each time point [*n* = 3 for eIF2B + eIF2(αP) + SFSV NSs at 100 nM in **c**, *n* = 4 for eIF2B + eIF2(αP) + SFSV NSs at 20, 40, 60 nM in (**c**), eIF2B + eIF2(αP) in (**d** and **e**), eIF2B + eIF2(αP) + SFSV NSs-c1-Ala-mut, eIF2B + eIF2(αP) + SFSV NSs-c1 + 2-Ala-mut in (**e**), and *n* = 5 for the rest of the experiments. *n* means the number of independent experiments]. Source data are provided as a Source Data file.

SFSV NSs exhibited tight binding to eIF2B, with a dissociation constant of 16.9 ± 2.8 nM measured by microscale thermophoresis (MST) (Fig. 2a). In our experimental settings, the dissociation constant between eIF2(αP) and eIF2B is 97.4 ± 15.0 nM (Supplementary Fig. 2a), showing that SFSV NSs binds to eIF2B more robustly than eIF2(αP). One feature of the interface between eIF2B and SFSV NSs is the abundance of aromatic residues on the SFSV NSs side. These residues form two clusters (clusters 1 [c1] and 2 [c2]) and bury spaces between the helices of the eIF2Bα subunit (Fig. 2b). The importance of these aromatic residues was examined by substitutions with alanine. The alanine substitutions in c2 (SFSV NSs-c2-Ala-mut; Y79A and F80A) weakened the interaction by about 6-fold and those in c1 (SFSV NSs-c1-Ala-mut; Y5A, F7A, and F33A) or in both clusters (SFSV NSs-c1 + 2-Ala-mut; Y5A, F7A, F33A, Y79A, and F80A) resulted in more than 100-fold reductions of the interaction (Fig. 2a).

The interactions mediated by the eIF2B subunits other than the α-subunit are limited to those mediated by eIF2Bδ R321, which interacts with T35 and H36 of SFSV NSs (Supplementary Fig. 2b). The alanine substitutions of these two residues only resulted in an approximately threefold increase of the dissociation constant (Supplementary Fig. 2c) and still retained relatively high affinity. This shows that the interaction between SFSV NSs and eIF2B is highly dependent on the α-subunit and the other subunits make modest contributions.

These results may suggest that the isolated eIF2Bα dimer could bind to SFSV NSs. However, we were unable to detect the binding between the isolated eIF2Bα dimer and SFSV NSs by either size exclusion chromatography or MST (Supplementary Fig. 2d, e). Interestingly, the structural comparison of eIF2Bαs in the isolated eIF2Bα dimer^24^ and in the eIF2B•SFSV NSs complex showed that the rotation angles of helix α3 differ, and thus the directions of the residues involved in the interaction with SFSV NSs also differ (Supplementary Fig. 2f). As the helix-α3 angle of the α-subunit in NSs-bound eIF2B is the same as that in apo eIF2B^14^, the assembly of eIF2Bα into the eIF2B decamer induces this rotation. This is likely to be the mechanism that allows SFSV NSs to bind exclusively to the α-subunit in the eIF2B decamer, but not to that in the isolated eIF2Bα dimer.

### SFSV NSs binding suppresses the inhibitory effect of eIF2(αP) on eIF2B

To understand the effect of the SFSV NSs binding on the eIF2(αP)-mediated eIF2B inhibition, we performed GDP exchange experiments. The results revealed that the guanine nucleotide exchange activity of eIF2B was inhibited in the presence of eIF2(αP), as previously described. The inclusion of SFSV NSs to the reaction suppressed this inhibitory effect of eIF2(αP) in a concentration-dependent manner (Fig. 2c). Although the suppression by ISRIB was only partial, as previously reported^14^, that by SFSV NSs was more robust and almost nullified the inhibitory effect of eIF2(αP) (Fig. 2c, d). This may reflect the difference in their eIF2B-binding modes, in which SFSV NSs physically covers the interface for eIF2(αP), but ISRIB does not. The suppression was weakened or canceled by the alanine substitutions of the aromatic clusters of SFSV NSs and their effects correlated well with their binding affinities to eIF2B (Fig. 2e). This indicated that (i) the suppression of the inhibitory effect of eIF2(αP) depends on the binding of SFSV NSs to eIF2B and (ii) the binding excludes eIF2(αP) from eIF2B, thus protecting eIF2B from the inhibitory action of eIF2(αP). In addition, these results demonstrated that the suppression strength of SFSV NSs can be controlled as intended, by introducing appropriate mutations of the eIF2B-interacting residues.

The currently proposed mechanism is apparently inconsistent with the previous observation that SFSV NSs does not interfere with eIF2(αP) binding to eIF2B^11^. Although the reason for this discrepancy is not clear, some fraction of eIF2B may bind one SFSV NSs molecule and one eIF2(αP) molecule simultaneously, through two distant interfaces around two symmetrically positioned eIF2Bα subunits.

### SFSV NSs-specific interaction site on eIF2B

Even though the eIF2B interfaces for eIF2(αP) and SFSV NSs overlap, they are not exactly identical. For example, the recognition of the C-terminal region of the helix α3 of eIF2Bα is unique to SFSV NSs (Supplementary Fig. 2g). We selected A47 of eIF2Bα, which resides at the interface for SFSV NSs and is not involved in the interaction with eIF2(αP), and mutated it to glutamate (predicted to clash with SFSV NSs). As expected, this mutation abrogated the interaction with SFSV NSs but not that with eIF2(αP) (Supplementary Fig. 2h). Consistently, SFSV NSs cannot suppress the inhibitory effect of eIF2(αP) on the guanine nucleotide exchange by this mutant eIF2B (Supplementary Fig. 2i).

Most of the eIF2Bα residues in this SFSV NSs-specific region are conserved in mammals (Supplementary Fig. 2j), but position 51, serine in human (*Homo sapiens*), is substituted in some species. Not all substitutions may be detrimental to SFSV NS binding, as this position is not strictly recognized, but substitutions of aromatic residues or arginine [as observed in mouse (*Mus musculus*) and horse (*Equus caballus*)] may cause steric clashes.

### Binding of SFSV NSs is compatible with the catalytic activity of eIF2B

**Fig. 3:**
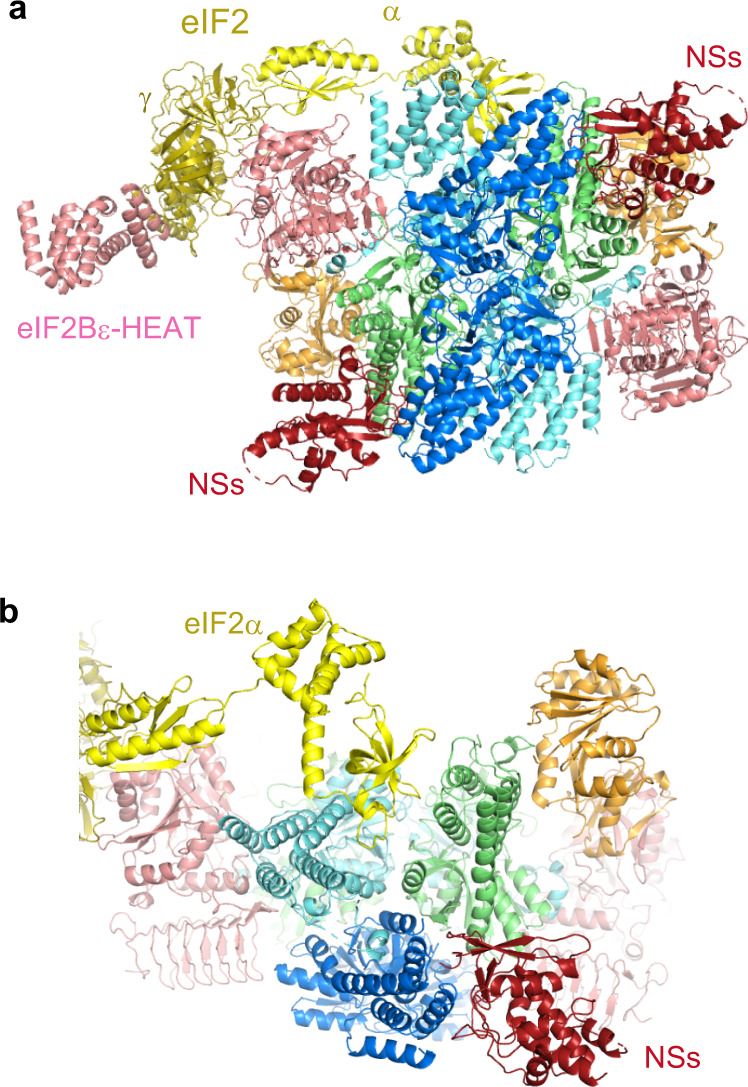
Cryo-EM structure of the human eIF2B•SFSV NSs•unphosphorylated eIF2 complex. **a** Overall structure of the human eIF2B SFSV NSs unphosphorylated eIF2 (one eIF2-bound) complex. eIF2B and SFSV NSs are color-coded as in Fig. 1. The eIF2α and eIF2γ subunits are colored yellow and olive, respectively. **b** Close-up view of the interfaces for SFSV NSs and eIF2α. These two molecules can bind eIF2B simultaneously and there is no direct interaction between them.

We also analyzed the mixture of human eIF2B, unphosphorylated eIF2, and SFSV NSs, and obtained the cryo-EM structures of the eIF2B•SFSV NSs•unphosphorylated eIF2 ternary complex (Fig. 3). Most of the ternary complex particles contained only one molecule of eIF2 (Fig. 3a), but small fractions of those containing two eIF2 molecules were also observed (Supplementary Fig. 3 and Supplementary Table 1). There are no notable structural differences in eIF2B or SFSV NSs between these two ternary complexes. These structures revealed that SFSV NSs and the unphosphorylated eIF2 can bind to eIF2B simultaneously and there is no direct interaction between them (Fig. 3b). The SFSV NSs binding does not seem to affect the unphosphorylated eIF2α binding, because SFSV NSs-bound eIF2B is able to accommodate eIF2 without major conformational changes. The only exception is the quite small closure of the interface for unphosphorylated eIF2 (Supplementary Fig. 3g), as observed in the comparison of the eIF2B•unphosphorylated eIF2 complex structure with the eIF2B apo structure^14^. The conformation of eIF2 is essentially the same as that in the eIF2B•unphosphorylated eIF2 complex structures and the switch 1 region in the nucleotide-binding eIF2γ subunit is widely open as well. Therefore, it seems to be trapped in the nucleotide-free state^3,4^ (Supplementary Fig. 3h). We also performed the guanine nucleotide exchange assay to determine the effect of SFSV NSs on the catalytic activity of eIF2B and found that NSs accelerates this activity (Supplementary Fig. 3i). As the interface for eIF2(αP) on eIF2B can accommodate unphosphorylated eIF2α in a similar manner^4^, the binding of SFSV NSs to eIF2B seems to block this non-catalytic interaction between unphosphorylated eIF2 and eIF2B, and thus contributes to this acceleration. However, other mechanisms such as positive allosterism cannot be ruled out. At the very least, we can conclude that the binding of SFSV NSs and the catalysis on unphosphorylated eIF2 can compatibly work.

### SFSV NSs attenuates the ISR in cellular contexts

**Fig. 4:**
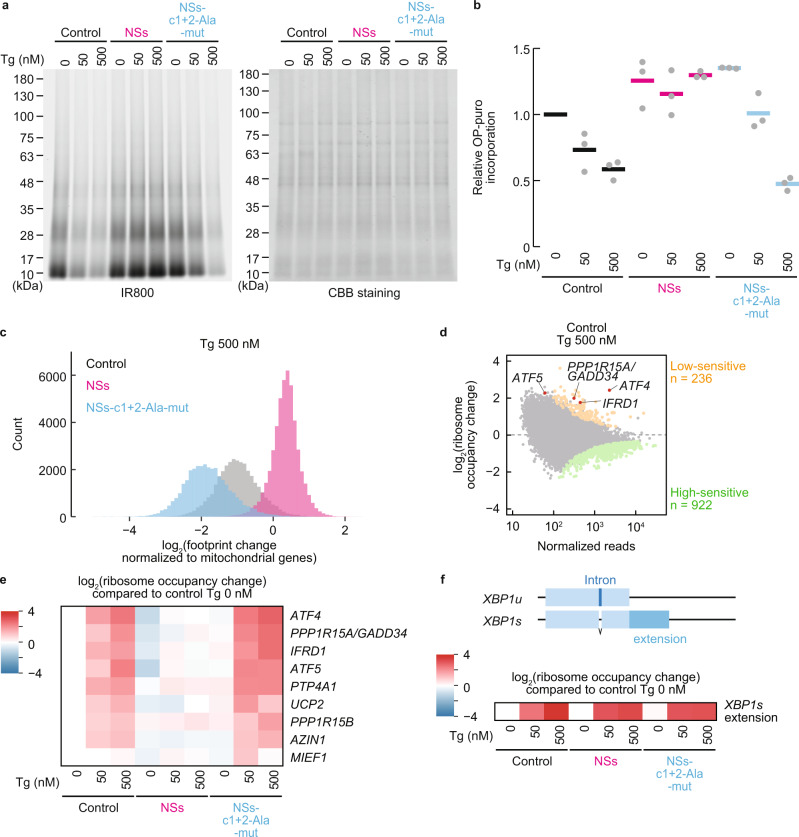
SFSV NSs suppresses translational impacts induced by thapsigargin. **a** Global protein synthesis rate measured by OP-puro labeling in control, SFSV NSs-expressing, or SFSV NSs-c1 + 2-Ala-mut-expressing cells. Cells were also treated with 50 or 500 nM Tg. Representative images of three biologically independent replicates of labeled nascent proteins (IR800 signal) and total protein with Coomassie Brilliant Blue (CBB) staining are shown. **b** Quantification of nascent proteins labeled with OP-puro, normalized by total protein in (**a**). Data of three replicates (points) and the means (bars) are shown. **c** Histogram of the number of transcripts along the footprint change in cells treated with 500 nM Tg. Data were normalized to the mean of footprint change of mitochondrial genome-encoded genes (used as internal spike-ins). Bin width is 0.1. **d** MA (M, log ratio; A, mean average) plot of the ribosome occupancy change in control vector-transfected cells treated with 500 nM Tg. High-sensitive and low-sensitive mRNAs (defined as false discovery rate [FDR] < 0.05) are highlighted. **e**, **f** Heatmap of ribosome occupancy changes on uORF-containing stress-resistant mRNAs (identified in ref. ^28^) (**e**) and *XBP1s* extension (**f**), compared to control vector-transfected cells treated with 0 nM Tg. The log_2_-fold change scales are shown at the color bars. Schematic representations of the *XBP1* gene are shown on the top of **f**. The intron in the *XBP1u* mRNA is spliced to produce the C-terminally extended protein upon ER stress. Source data are provided as a Source Data file.

The inhibition of eIF2B by eIF2(αP) results in a global reduction of protein synthesis. To observe the cellular effect of SFSV NSs on overall translation during stress, human embryonic kidney (HEK) 293 cells were treated with thapsigargin (Tg) to induce endoplasmic reticulum (ER) stress, activate PERK, and lead to the eIF2α phosphorylation and consequently the ISR. The Tg treatment shut off global translation, as monitored by the metabolic labeling of newly synthesized proteins with *O*-propargyl-puromycin (OP-puro)^25^, in a dose-dependent manner (Fig. 4a, b). Strikingly, the ectopic expression of wild-type SFSV NSs (Supplementary Fig. 5a) rescued the translation repression induced by Tg, whereas, in stark contrast, eIF2B-unbound mutant SFSV NSs (c1 + 2-Ala-mut) lost this activity (Fig. 4a, b).

The SFSV NSs-mediated recovery of translation repression in the ISR was also ensured in individual transcripts. To obtain an overview of the landscape of translational output in the Tg-induced ISR and its recovery by SFSV NSs, we employed ribosome profiling^26^ (for the quality of ribosome profiling data and determination of A-site offsets, see “Methods”, Supplementary Fig. 4, and Supplementary Table 2). To assess the global translation change, we used mitochondrial ribosome footprints as an internal spike-in^27^. Congruent with the OP-puro results, the Tg-mediated translation repression across the transcriptome was recovered by wild-type SFSV NSs, but not by mutant SFSV NSs (Fig. 4c and Supplementary Fig. 5b, c).

To further investigate the net alteration of translation over the transcriptome, which may be the ultimate consequence of ER stress^1^, we evaluated the relative ribosome occupancy on mRNA by the ribosome footprint abundance normalized by RNA sequencing (RNA-Seq) (Supplementary Table 3). The ribosome occupancy changes in the ISR were not uniform among the transcripts and a group of mRNAs, such as ribosomal protein mRNAs, were highly sensitive to Tg (Fig. 4d and Supplementary Fig. 5d). The translational recovery of these high-sensitivity mRNAs was accentuated upon wild-type SFSV NSs expression (Supplementary Fig. 5e).

Along with the suppression of protein synthesis, the phosphorylation of eIF2α simultaneously entails the translational activation of a subset of mRNAs to respond to the stress^5^. This transcript-selective activation is associated with upstream open reading frames (uORFs) in the 5′-untranslated region, which normally trap the scanning ribosome to block the complex from reaching the main ORF further downstream. The reduced availability of the eIF2•GTP•Met-tRNA_i_ ternary complex evokes “leaky” scanning to skip the uORFs and then drives translation from the downstream main ORF. *ATF4*, a stress-inducible transcription factor, is a remarkable example of translational stimulation through uORFs^1^. Indeed, upon Tg treatment, the translation from the main *ATF4* ORF was increased (Fig. 4d, e and Supplementary Fig. 5f). In contrast, wild-type SFSV NSs expression did not activate *ATF4* translation, even with Tg treatment (Fig. 4e and Supplementary Fig. 5f). In addition to *ATF4*, uORF-bearing mRNAs, including *PPP1R15A* (also known as *GADD34*, coding a growth arrest and DNA damage inducible protein), *IFRD1*, *ATF5*, *PTP4A1*, *UCP2*, *PPP1R15B*, and *AZIN1*, were susceptible to similar translation activation within the ISR^28^ (Fig. 4d, e). SFSV NSs also inhibited the activation of these mRNAs (Fig. 4e). On the other hand, eIF2B-unbound mutant SFSV NSs did not phenocopy the impact of wild-type SFSV NSs (Fig. 4e). We also note that the translation changes were not associated with the mRNA abundance changes (Supplementary Fig. 5g).

In addition to PERK activation and subsequent eIF2α phosphorylation, ER stress also drives alternative branches of the stress response pathway, such as ERN1 (also known as IRE1) activation and the subsequent *XBP1* mRNA splicing^29,30^, generating the *XBP1s* (spliced) mRNA with an extended ORF. The Tg-induced footprint accumulation in the extended ORF demonstrated the proper splicing of the mRNA and IRE1 activation even under the conditions with SFSV NSs expression (Fig. 4f and Supplementary Fig. 6a), and thus confirmed the high specificity of SFSV NSs in antagonizing the eIF2(αP) branch, but not the IRE1 branch of the ER stress response. We also examined the effect of SFSV NSs on autophagy induced by the ER stress by probing for the MAP1LC3B (also known as LC3B)-phosphatidylethanolamine conjugate (or LC3B-II), as its emergence is a hallmark of autophagy induction. Given that ATF4 activation drives autophagy-related gene expression^1^, the block may hamper the autophagy, in theory. However, we did not detect a clear difference in LC3B-II accumulation upon SFSV NSs expression (Supplementary Fig. 6b, c). In our HEK293 cells, XBP1s, which was not affected by SFSV NSs, may compensate for the loss of ATF4 for autophagy induction^31^.

Taken together, our data demonstrate the clear correspondence between the cellular functions of SFSV NSs and its mutants and their structural and biochemical propensities.

### SFSV NSs protects rat hippocampal neurons and human iPS cell-derived motor neurons from ISR-inducible stress

**Fig. 5:**
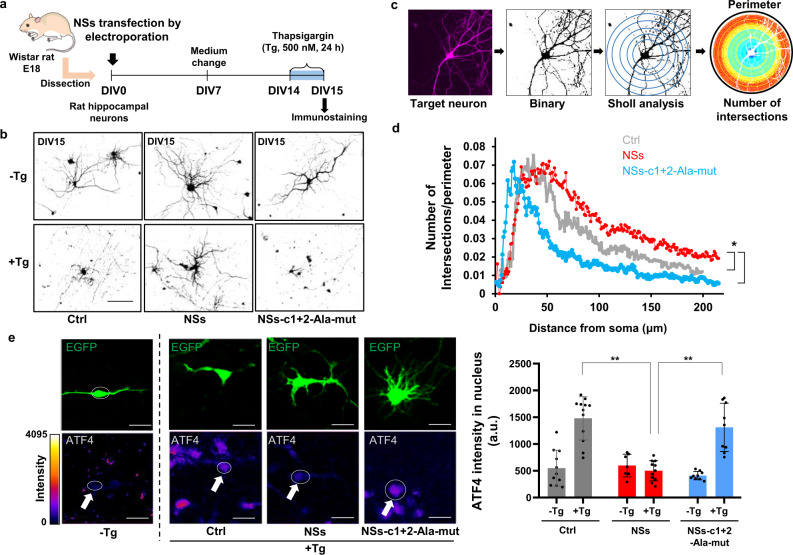
SFSV NSs in rat primary hippocampal neurons attenuates the ISR induction and protects the neuronal morphology. **a** Schematic illustration of thapsigargin treatment (500 nM) for rat primary hippocampal neurons. The neurons were transfected with the control plasmid, the SFSV NSs-expression plasmid, or the SFSV NSs-c1 + 2-Ala-mut-expression plasmid by electroporation. **b** Representative images of hippocampal neurons with or without Tg treatment after 24 h. Tg treatment shortened the neurites in the control and NSs-c1 + 2-Ala-mut-expressing cells, whereas NSs counteracted Tg. Scale bar = 100 µm. **c** Schematic representation of the Sholl analysis. **d** The Sholl analysis revealed that NSs protected the arborization of neurons. *n* = 5. **e** NSs downregulated ATF4 expression induced by Tg (500 nM, 3 h), as compared to the control and NSs-c1 + 2-Ala-mut. *n* = 3 (seven targeted neurons), *n* means the number of independent experiments. *p < 0.05, a.u., arbitrary units. Scale bar = 10 µm. The 12-bit scale of fluorescent intensity is shown as color coded. **d** One-way ANOVA with Tukey’s multiple comparisons test. **e** Two-way ANOVA with Tukey’s multiple comparisons test. Error bars ± SD. **a**, **b** DIV, days in vitro. Source data are provided as a Source Data file.

Considering that ISRIB shows beneficial effects in various neuropathological models^16–19^, it is tempting to speculate that SFSV NSs also effectively works in such conditions. To assess the neuroprotective activity of SFSV NSs under the ISR-inducible stress conditions, we prepared primary cultures of hippocampal neurons from rat embryos, whose eIF2Bα is expected to be compatible with SFSV NSs (Fig. 5 and Supplementary Fig. 2j). The ISR-inducing Tg treatment of rat primary hippocampal neurons decreased the length of their neurites (Fig. 5a, b). To quantify the arborization of neurites, a Sholl analysis was performed (Fig. 5c). The expression of SFSV NSs significantly attenuated the neurite degradation triggered by Tg, whereas that of the mutant SFSV NSs (c1 + 2-Ala-mut) did not indicate neuroprotective activity (Fig. 5b, d). Immunostaining of ATF4 in the control neurons demonstrated that the Tg treatment significantly upregulated its expression (Fig. 5e). This neurodegeneration, along with the upregulation of ATF4, indicated that the Tg treatment sufficiently triggered ISR through the stress^32^. Consistent with the phenotypic effect, wild-type SFSV NSs significantly suppressed the induction of ATF4 expression, in contrast to the mutant SFSV NSs (Fig. 5e).

**Fig. 6:**
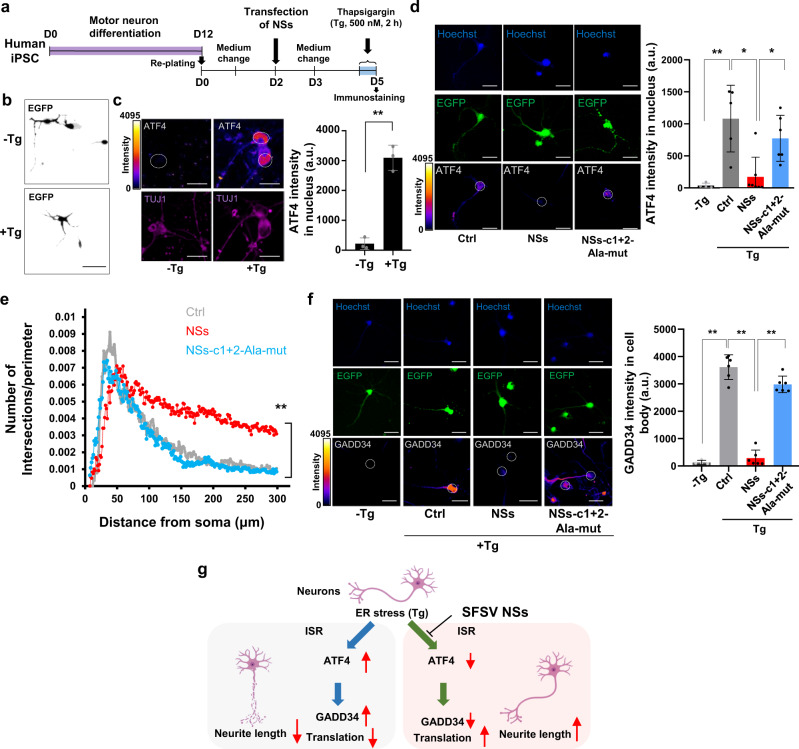
SFSV NSs in human iPS cell-derived motor neurons attenuates the ISR induction and exhibits neuroprotective effects. **a** Motor neuron differentiation from human iPS cells and the timing of NSs transfection and drug administration. After motor neurons were replated onto glass coverslips, they were transfected with the control plasmid, the SFSV NSs-expression plasmid, or the SFSV NSs-c1 + 2-Ala-mut-expression plasmid by Lipofectamine 3000 for 2 days. After additional 3 days, the neurons were treated with thapsigargin (Tg; 500 nM) for 2 h, followed by fixation and immunostaining of ATF4. **b** Representative images of motor neurons with or without Tg treatment. Scale bar = 50 µm. **c** Tg significantly induced the stress response, as represented by ATF4 upregulation in motor neurons. *n* = 3. Scale bar = 10 µm. **p < 0.01. **d** ATF4 intensity in control, SFSV NSs-expressing, and SFSV NSs-c1 + 2-Ala-mut-expressing motor neurons after Tg treatment. In SFSV NSs-expressing motor neurons, ATF4 was significantly downregulated, whereas mutant SFSV NSs expression did not alter ATF4 protein levels as compared to the control. *n* = 3. *p < 0.05. Scale bar = 10 µm. **e** Sholl analysis in motor neurons. NSs expression increased the length of axons as compared to the control and NSs-c1 + 2-Ala-mut expression. *n* = 5. **f** ISR triggered a significant increase of immunoreactivity to the GADD34 antibody. In addition, NSs treatment significantly decreased the expression level of GADD34, whereas NSs-c1 + 2-Ala-mutant did not alter the GADD34 level as compared to the control. *n* = 5. Scale bar = 10 µm. **g** Illustration of ISR induction and NSs effect upon ER stress in neurons. *n* means the number of independent experiments *p < 0.05; **p < 0.01. **h** Student’s *t*-test; **d**–**f** One-way ANOVA with Tukey’s multiple comparisons test. Error bars ±SD. **c**, **d**, **f** a.u., arbitrary units. The 12-bit scale of fluorescent intensity is shown at color coded. Source data are provided as a Source Data file.

The effect of SFSV NSs was further confirmed with human iPS cell-derived motor neurons^33^ (Fig. 6 and Supplementary Fig. 7a). Tg treatment for 2 h increased ATF4 in motor neurons to the similar level as in rat neurons (Fig. 6c). SFSV NSs efficiently suppressed the induction of ATF4 even with the Tg treatment, but the mutant SFSV NSs did not (Fig. 6d). SFSV NSs also promoted the axon lengthening and increased the density of elongated axons, as characterized by the Sholl analysis, as compared to the control and mutant SFSV NSs (Fig. 6e). In addition, ISR by Tg triggered a significant increase of immunoreactivity to the antibody against GADD34, a transcriptional target of ATF4^1^ (Fig. 6f). In the same manner as the ATF4 regulation, SFSV NSs significantly decreased the expression level of GADD34 along with ATF4 downregulation, whereas the NSS-c1 + 2-Ala-mutant did not alter the GADD34 level as compared to the control. Although GADD34 serves as a negative-feedback-loop regulator for the eIF2α-ATF4 pathway, it is also known that GADD34 induces cell death through the suppression of Akt^34^. Consistently, under stressed conditions, the autophagic function is highly activated, causing the degradation of mitochondrial and axonal proteins^35^. Although the ratio of LC3B-II/LC3B-I was not changed by the SFSV NSs treatment in HEK293 cells (Supplementary Fig. 6b, c), we assumed that the SFSV NSs treatment mediating ATF4 downregulation can potentially modulate autophagy to maintain homeostasis, especially in axons.

Taken together, in neurons SFSV NSs can suppress the ISR mediated by ATF4 and GADD34, thus providing a neuroprotective effect (Fig. 6g). Of note, Tg treatments longer than 24 h caused serious degradation characterized by broken axons and detached cells. From a technical perspective, our experimental conditions are not appropriate to demonstrate the reversal of chronic neurodegenerative conditions, since long-term Tg treatment ultimately results in critical damage of the neurons (Supplementary Fig. 7b). We hope our rescue strategy with NSs not only alleviates the stress response in the short-term but also helps to recover or prevent neurodegeneration for the long term. This will be addressed in future experiments.

### SFSV NSs is a promising therapeutic ISR inhibitor

We have solved the cryo-EM structures of the eIF2B•SFSV NSs complex and the eIF2B•SFSV NSs•unphosphorylated eIF2 ternary complex, and analyzed the catalytic effect of SFSV NSs binding to eIF2B upon its inhibition by eIF2(αP). The binding of SFSV NSs sterically blocks the inhibitory interaction between eIF2B and eIF2(αP), whereas it is compatible with the catalytic interaction between eIF2B and unphosphorylated eIF2 (Figs. 1 and 3). As a result, eIF2B suppresses the inhibitory effect of eIF2(αP) and continues to catalyze nucleotide exchange on residual unphosphorylated eIF2, even in the presence of high levels of eIF2(αP) (Fig. 2). This is likely to be the molecular mechanism by which SFSV enables viral protein synthesis against the PKR-derived host cell defense.

The suppression of the ISR by SFSV NSs was observed in several kinds of animal cells: HEK293 cells, rat hippocampal neurons, and human iPS-derived motor neurons (Figs. 4–6). In parallel with us, Schoof et al.^36^ have confirmed that SFSV NSs can suppress the ISR irrespective of any activating kinases. Interestingly, the supplementation of SFSV NSs to neural cells exhibited neuroprotective effects, along with the downregulation of ATF4 and GADD34, under the ISR-inducible stressed conditions (Figs. 5 and 6). Our newly identified direct blocking mechanism of SFSV NSs against eIF2(αP) on eIF2B was more effective than the allosteric antagonism by ISRIB^14,15^ (Fig. 2d). Therefore, these observations raise the possibility that, similar to ISRIB, SFSV NSs could also show beneficial effects in neuropathological conditions. Future studies on the in vivo effects of SFSV NSs, administered as a drug in the respective animal models, may reveal the potential therapeutic utility of this strong ISR inhibitor.

## Methods

### Protein preparation

The SFSV NSs gene was cloned into the pET-28a vector (Novagen) and expressed with a C-terminal His-tag in the T7 Express competent *E. coli* strain (NEB). The cells were grown at 37 °C in Luria-Bertani (LB) medium supplemented with 0.2% (w/v) glucose. After the addition of 0.3 mM isopropyl-β-d-thiogalactopyranoside (IPTG) at *A*_600_ = 0.5, the cells were further grown overnight at 18 °C. The collected cells were lysed in lysis buffer [20 mM HEPES-KOH buffer pH 7.5, containing 150 mM KCl, 5% (v/v) glycerol, and 1 mM dithiothreitol (DTT)] supplemented with protease inhibitors. After centrifugation, the supernatant was purified with cOmplete His-Tag Purification Resin (Roche) and by chromatography on a HiTrap Q HP column (GE Healthcare) with a 150‒500 mM KCl gradient. The sample was further purified by chromatography on a Superdex 200 column (GE Healthcare) equilibrated with lysis buffer. The SFSV NSs mutants were also purified in the same manner.

Human eIF2B and eIF2 were recombinantly expressed and purified^4,14^. eIF2B was prepared by mixing separately-purified eIF2Bα and eIF2Bβγδε. Both of the *E. coli* cells expressing eIF2Bα and the cells co-expressing eIF2Bβγδε were grown and lysed as above. eIF2Bα was purified with cOmplete His-Tag Purification Resin and flowed through Q Sepharose Fast Flow (GE Healthcare) and SP Sepharpose Fast Flow (GE Healthcare). After dialysis against lysis buffer and cleavage by HRV 3C protease, the sample was flowed through cOmplete His-Tag Purification Resin, and further purified by chromatography on a Superdex 200 column equilibrated with lysis buffer. eIF2Bβγδε was purified with Ni Sepharose 6 Fast Flow (GE Healthcare), and by chromatography on a HiTrap Heparin HP column (GE Healthcare) with a 0.15‒1 M KCl gradient. After dialysis against lysis buffer and cleavage by HRV 3C protease, the sample was supplemented with 10 mM imidazole and flowed through Ni Sepharose 6 Fast Flow. eIF2Bβγδε was mixed with an excess of eIF2Bα and further purified on a Sephacryl S-300 column (GE Healthcare) equilibrated with lysis buffer.

For the preparation of eIF2, human eIF2 subunits and human CDC123 were co-expressed in FreeStyle293-F cells (Thermo Fisher Scientific) cultured in FreeStyle293 Expression Medium (Thermo Fisher Scientific) at 37 °C in 8% CO_2_ atmosphere. The cells were lysed in buffer A [20 mM MES-KOH buffer pH 6.0, containing 150 mM KCl, 1 mM MgCl_2_, 10% (v/v) glycerol, and 5 mM 2-mercaptoethanol] supplemented with 20 mM imidazole, 0.5 mM EDTA, 0.1% (v/v) Triton X-100, and protease inhibitors. After centrifugation, the supernatant was applied to a HisTrap HP column (GE Healthcare) equilibrated with buffer A supplemented with 20 mM imidazole and eluted with a 20‒500 mM imidazole gradient. The sample was applied to a HiTrap SP HP column (GE Healthcare) equilibrated with buffer A and eluted with a 200‒640 mM KCl gradient. After dilution with buffer B [20 mM HEPES-KOH buffer (pH 7.5) containing 100 mM KCl, 0.1 mM MgCl_2_, 10% (v/v) glycerol, and 1 mM DTT], the sample was applied to a HiTrap Heparin HP column equilibrated with buffer B, and eluted with a 0.2‒1 M KCl gradient and further purified on a Superdex 200 column equilibrated with buffer B. The plasmids used for the preparation are listed in Supplementary Table 4.

### Cryo-EM data collection and image processing

For the cryo-EM analyses, eIF2B and SFSV NSs were mixed at a molar ratio of 1 : 4, diluted to 60 nM for eIF2B and 240 nM for SFSV NSs, and supplemented with 0.06% (w/v) digitonin. For the eIF2B•SFSV NSs•unphosphorylated eIF2 ternary complex, eIF2B, SFSV NSs, and eIF2 were mixed at a molar ratio of 1 : 3 : 4, diluted to 50 nM for eIF2B, 150 nM for SFSV NSs, and 200 nM for eIF2, and supplemented with 0.06% (w/v) digitonin. The grids were prepared using a Vitrobot Mark IV (FEI) at 4 °C and 100% humidity. The samples (3 μl) were loaded onto Quantifoil R1.2/1.3 300 mesh copper grids (Quantifoil) covered with an in-house prepared amorphous carbon layer, incubated for 30 s, blotted for 3 s, and plunged into liquid ethane.

The datasets were collected with a Krios G4 transmission electron microscope (FEI) operated at 300 kV, using a K3 direct electron detector (Gatan) operated in the CDS-counting mode (0.829 Å/pixel), running at the RIKEN Center for Biosystems Dynamics Research in Yokohama, Japan. Automatic data collection was performed using EPU 2.9 software (FEI). The total numbers of collected images were 12,341 for the eIF2B•SFSV NSs complex sample and 13,858 for the eIF2B•SFSV NSs•unphosphorylated eIF2 complex sample. The collected images were fractionated to 50 frames, with a total dose of ~50 e^−^/Å^2^.

Processing of cryo-EM data was performed with RELION-3.1^37^. The movie frames (from 2nd frame to 50th frame) were aligned with MotionCor2 (RELION’s own implementation) and the contrast transfer function (CTF) parameters were estimated with CTFFIND-4.1^38^. Particles were automatically picked using template-free Laplacian-of-Gaussian filters (diameters from 150 to 300 Å).

For the eIF2B•SFSV NSs complex sample, the 3,413,435 automatically picked particles were extracted with two-fold binning (1.658 Å/pixel). After splitting the particles into three subsets, two-dimensional (2D) classification was performed twice. For three-dimensional (3D) classification, good classes from the three subsets were merged and split again into three subsets, and a low-pass filtered (40 Å) map calculated from the crystal structure of *Schizosaccharomyces pombe* eIF2B (PDB: 5B04)^39^ was used as the reference map. After the classification, the particles in the good classes from the three subsets that contained the SFSV NSs-like protrusion from the density of eIF2B (899,315 particles) were joined and re-extracted without rescaling (0.829 Å/pixel) and subjected to the 3D refinement, Bayesian polishing, and CTF refinement, and the 3D refinement again. The refined particles were subjected to 3D classification again and the junk particles were discarded. Afterwards, the map was aligned and refined with C2 symmetry. The final number of particles is 888,479.

For the eIF2B•SFSV NSs•unphosphorylated eIF2 complex sample, the 4,482,492 automatically picked particles were similarly extracted, split into subsets, and subjected to 2D classification. For 3D classification, a low-pass filtered (40 Å) map of eIF2B SFSV NSs was used as the reference map. The classes containing eIF2B, SFSV NSs, and additional density (615,804 particles) were merged into one dataset and classified again. The classes containing the eIF2-like density (125,080 particles) were re-extracted without rescaling, and subjected to the 3D refinement, Bayesian polishing, and CTF refinement, and the 3D refinement again. The refined particles were subjected to 3D classification again and separated into the class containing 1 eIF2 molecule (114,137 particles) and the class containing 2 eIF2 molecules (8106 particles).

To build a model of the eIF2B•SFSV NSs complex, the human eIF2B moiety in the cryo-EM structure of the eIF2B•phosphorylated eIF2α complex at 3.0 Å resolution (PDB: 6O9Z)^3^ was used as the initial model. At first, the model of eIF2B was manually fitted into the maps using UCSF Chimera-1.15^40^. Map sharpening and model refinement were performed in PHENIX 1.19.2^41^ and the models were further refined manually with Coot-0.9.4.1^42^. The model for SFSV NSs was built automatically with the Buccaneer version 1.6.9^43^ in the CCP-EM v1.5.0^44^, and manually with Coot. For the eIF2B SFSV NSs unphosphorylated eIF2 complex, the eIF2B SFSV NSs complex structure, eIF2, and the HEAT domain of the eIF2Bε subunit from the cryo-EM structure of the eIF2B unphosphorylated eIF2 complex at 3.0 Å resolution (PDB: 6O85)^3^ were manually fitted into the maps with USCF Chimera, and refined with PHENIX and Coot. The models were also validated with MolProbity 4.5.1^45^.

The statistics for image processing and refinement are summarized in Supplementary Table 1.

### Microscale thermophoresis

Purified SFSV NSs was dialyzed against measurement buffer [20 mM HEPES-KOH buffer pH 7.4, containing 150 mM KCl and 5 mM MgCl_2_] overnight and supplemented with 0.05% (v/v) Tween-20 after dialysis. The fluorescently labeled SFSV NSs protein was prepared by mixing equal volumes of 200 nM SFSV NSs and 150 nM HIS Lite OG488-Tris NTA-Ni Complex (AAT Bioquest). Fluorescently labeled eIF2 was also prepared in the same manner. For the phosphorylation of eIF2, PKR is concentrated to 2 mg/ml and activated by incubating with 1 mM ATP at 30 °C for 1 h at first^4^. Then, 500 μM ATP and ~50 nM activated PKR were added to the solution containing eIF2, and incubated for 5 min at 23 °C. The purified eIF2B or eIF2Bα dimer (5.0–6.8 μM) was prepared by dialysis against measurement buffer and supplementation with 0.05% (v/v) Tween-20. MST was measured at 23 °C with a Monolith NT.115 (Nanotemper), according to the manufacturer’s protocol. Each experiment was repeated three times and dissociation constants were calculated using MO.Affinity Analysis v2.2.7 (Nanotemper).

### Size exclusion chromatography

Purified eIF2Bα dimer was mixed with purified SFSV NSs or with lysis buffer (30 μM for the eIF2Bα dimer, 100 μM for SFSV NSs) and applied to a Superdex 200 column equilibrated with lysis buffer.

### Guanine nucleotide exchange assay

The eIF2B guanine nucleotide exchange activity was measured by the previous method^14^ with some modifications. Purified eIF2(αS51A) was loaded with BODIPY-FL-GDP (Invitrogen) in loading buffer [20 mM HEPES-KOH buffer pH 7.4 containing 150 mM KCl, 1 mM DTT, 0.05 mg/ml bovine serum albumin (BSA), and 0.01% (v/v) Triton X-100] at room temperature. After supplementation with 2 mM MgCl_2_, the unbound fluorescent dye was removed and the buffer was exchanged to assay buffer [20 mM HEPES-KOH buffer pH 7.4 containing 150 mM KCl, 2 mM MgCl_2_, 1 mM DTT, 0.05 mg/ml BSA, 0.01% (v/v) Triton X-100, and 1.5 mM GDP] using ProbeQuant G-50 Micro Columns (Cytiva). eIF2(αP) was prepared using PKR and buffer exchange was performed as described above. The proteins were premixed in a 384-well Low Volume Black Round Bottom Polystyrene NBS Microplate (Corning) at final concentrations of 150 nM fluorescently labeled eIF2(αS51A), 0 or 1.5 μM eIF2(αP), 0‒200 nM SFSV NSs, and 0 or 200 nM ISRIB (Sigma-Aldrich), and reactions were started by the addition of 0, 5, or 40 nM eIF2B into the mixture at 25 °C. The fluorescence signal was read every 20 s by an EnVision 2104 plate reader (PerkinElmer), with excitation at 485 nm and emission at 535 nm, and each experiment was repeated three to five times.

### Plasmid construction

#### pI.18-3xFLAG and SFSVNSs-c1 + 2-Ala-mut-3xFLAG

To construct pI.18-3xFLAG and pI.18-SFSVNSs-c1 + 2-Ala-mut-3xFLAG, the SFSV NSs gene in pI.18-SFSVNSs-3xFLAG^11^ was either removed or replaced with a PCR-amplified DNA fragment encoding the mutated SFSV NSs gene, respectively.

### Nascent peptide labeling by OP-puro

HEK293 T-REx cells (Thermo Fisher Scientific) were cultured in a 24-well plate with Dulbecco’s modified Eagle medium (DMEM) + GlutaMAX-I (Thermo Fisher Scientific, 10566016) supplemented with 10% fetal bovine serum (FBS) (Sigma, F7524) at 37 °C in a 5% CO_2_ atmosphere, and transfected with 0.3 µg of the pI.18-3xFLAG, pI.18-SFSVNSs-3xFLAG, or pI.18-SFSVNSs-c1 + 2-Ala-mut-3xFLAG plasmid, using the TransIT-293 Reagent (Mirus, MIR2704). After 48 h incubation, the cells were treated with Tg (Nacalai Tesque) and 20 μM OP-puro (Jena Bioscience, NU-931-05) for 30 min at 37 °C. OP-puro labeling was performed as described previously^46^. Briefly, cells were washed with 0.5 ml phosphate-buffered saline (PBS) and lysed in 60 µl of lysis buffer (20 mM Tris-HCl pH 7.5, 150 mM NaCl, 5 mM MgCl_2_, and 1% Triton X-100). The lysate was incubated with 1 μM IRDye 800CW Azide (LI-COR Bioscience) for 30 min at 25 °C, using a Click-iT Cell Reaction Buffer Kit (Thermo Fisher Scientific, C10269). After the unreacted azide was removed by MicroSpin G-25 Columns (Cytiva, 27532501), the peptides were separated by sodium dodecyl sulfate–polyacrylamide gel electrophoresis (SDS-PAGE), followed by the acquisition of the IR800 signal by ODYSSEY CLx (LI-COR Biosciences). Subsequently, the total protein on the gel was stained by EzStain AQua (ATTO, AE-1340) for normalization. The signals of proteins ranging from 10 to 180 kDa were quantified and the background signal from a blank lane was subtracted.

### Western blotting

For Supplementary Fig. 5a, lysates for ribosome profiling were subjected to SDS-PAGE and immunoblotting. For Supplementary Fig. 6b, cells cultured in a 24-well plate were transfected with the pI.18-3xFLAG, pI.18-SFSVNSs-3xFLAG, or pI.18-SFSVNSs-c1 + 2-Ala-mut-3xFLAG plasmid, as described in the “Nascent peptide labeling by OP-puro” section. After 48 h incubation, the cells were treated with Tg for 0, 2, 4, and 6 h at 37 °C and lysed in 100 µl of RIPA buffer (Nacalai Tesque, 16488-34) with Protease Inhibitor Cocktail (Nacalai Tesque, 25955-24) and Halt Phosphatase Inhibitor (Thermo Fisher Scientific, 78428). The cleared lysates were subjected to SDS-PAGE and immunoblotting. Anti-LC3B (Abcam, ab48394, 1 : 1000), anti-FLAG M2 (Sigma, F3165, 1 : 1000), anti-β-actin mAb 6D1 (Medical & Biological Laboratories [MBL], M177-3, 1 : 1000), and anti-β-actin pAb (MBL, PM053, 1 : 1000) were used as primary antibodies. IRDye 800CW anti-rabbit IgG (LI-COR, 926-32211, 1 : 10,000), IRDye 680RD anti-mouse IgG (LI-COR, 925-68070, 1 : 10,000), and IRDye 680RD anti-rabbit IgG (LI-COR, 926-68071, 1 : 10,000) were used as secondary antibodies. Images were acquired with an ODYSSEY CLx (LI-COR) imaging system.

### Ribosome profiling and RNA-Seq

#### Library preparation

HEK293 T-REx cells were cultured in 10 cm dishes with DMEM + GlutaMAX-I supplemented with 10% FBS at 37 °C in a 5% CO_2_ atmosphere, and transfected with 6 µg of the pI.18-3xFLAG, pI.18-SFSVNSs-3xFLAG, or pI.18-SFSVNSs-c1 + 2-Ala-mut-3xFLAG plasmid, using the TransIT-293 Reagent. After 48 h incubation, the cells were treated with Tg for 30 min at 37 °C. We set up two replicates of Tg non-treated samples in each plasmid transfection and others with a single replicate (Supplementary Tables 2 and 3), due to cost constraints. This experimental design should still have enough statistical power with the use of the differential expression analysis tool DESeq2^47^ (see below for details).

The ribosome profiling library was prepared according to the protocol described previously^48,49^. Briefly, cells were washed with 5 ml PBS and lysed in 600 µl of lysis buffer with 1 mM DTT, 100 µg/ml cycloheximide, and 100 µg/ml chloramphenicol. Lysates were treated with 15 U of Turbo DNase (Thermo Fisher Scientific, AM2238) and cleared by centrifugation at 20,000 × *g* for 10 min at 4 °C. The RNA concentrations in the lysates were measured with a Qubit RNA BR Assay Kit (Thermo Fisher Scientific, Q10210). A lysate containing 10 µg of RNA was digested with 20 U of RNase I (Lucigen, N6901K) for 45 min at 25 °C and ultracentrifuged for 1 h at 100,000 r.p.m. (408,800 × *g*) at 4 °C, using Optima MAX-TL and a TLA-110 rotor (Beckman Coulter). The RNA in the ribosome pellet was extracted with the Trizol reagent (Thermo Fisher Scientific, 15596018) and a Direct-zol RNA microprep kit (Zymo Research, R2060), and subjected to PAGE. Ribosome-protected RNA fragments ranging from 17 to 34 nt were excised from the gel. The purified RNA fragments were dephosphorylated with T4 polynucleotide kinase (New England Biolabs, M0201) for 1 h at 37 °C and ligated with a pre-adenylated linker DNA (Eurofins, 5′-Phos-NNNNNIIIIITGATCGGAAGAGCACACGTCTGAA-ddC-3′, where Phos indicates 5′-phosphorylation, Ns indicate a random sequence for eliminating PCR-duplicated reads, Is indicate the index sequence for multiplexing, and ddC indicates 2′,3′-dideoxycytidine), using T4 RNA Ligase 2 and truncated KQ (New England Biolabs, M0373) for 3 h at 22 °C in the presence of 15% PEG-8000. The linker-ligated RNA was extracted by PAGE purification, treated with the Ribo-Zero Gold rRNA Removal Kit in the TruSeq Stranded Total RNA kit (Illumina, 20020598), and reverse-transcribed by Protoscript II (New England Biolabs, M0368) for 30 min at 50 °C with a primer (Eurofins, 5′-Phos-NNAGATCGGAAGAGCGTCGTGTAGGGAAAGAG-iSp18-GTGACTGGA GTTCAGACGTGTGCTC-3′, where iSp18 indicates a hexa-ethyleneglycol spacer). The template RNA was hydrolyzed with 1 M NaOH. The PAGE-purified cDNA was circularized using CircLigaseII ssDNA ligase (Epicentre, CL9025K) for 1 h at 30 °C, and PCR-amplified with the primers (Hokkaido System Science, 5′-AATGATACGGCGACCACCGAGATCTACACTCTTTCCCTACACGACGCTC-3′ and 5′-CAAGCAGAAGACGGCATACGAGATJJJJJJGTGACTGGAGTTCAGACGTGTG-3′, where Js indicate the 6-mer index sequence for Illumina sequencing).

For RNA-Seq, RNA was extracted from the same lysate used for ribosome profiling with Trizol LS (Thermo Fisher Scientific, 10296-010) and a Direct-zol RNA microprep kit (Zymo Research, R2060), and 0.5 µg of the RNA was used for RNA-Seq library preparation with a TruSeq Stranded mRNA Library Prep Kit (Illumina). The libraries were sequenced on a HiSeq 4000 System (Illumina).

#### Data analysis

Sequence data were processed as previously described^48^ with the following modifications. Data collection was performed using Illumina Casava 1.8 software. Read quality filtering and adapter trimming were performed with Fastp^50^. After removing the non-coding RNA-mapped reads, the remaining reads were aligned to the human genome hg38 and assigned to the GENCODE Human release 32 reference obtained from UCSC Genome Browser (https://genome.ucsc.edu/index.html), using STAR 2.7.0a^51^. The numbers of reads after each step are summarized in Supplementary Tables 2 and 3. For mitochondrial genes, we mapped reads after non-coding RNA removal to the custom-made transcript sequences and annotations.

The offsets of the A-site were determined according to the metagene analysis for the 5′-end of footprints, using a custom script (Supplementary Fig. 4). For cytoplasmic ribosomes, 15 for 20–22 and 25–30 nt were used because the 5′-end of the peak, which represents 80S on the AUG start codon on the P-site, was at −12 nt from the start codons (Supplementary Fig. 4a, b). For mitochondrial ribosomes, 14 for 27–28 nt and 15 for 29–32 nt were used based on following observation: the fraction of 31 nt footprints was the maximum for all fragments mapped to mitochondrial transcripts (Supplementary Fig. 4c). In the plot of the 5′-end of footprints on leaderless transcripts (e.g., *MT-CO2* or *MT-ND4L*), 19 nt footprints were mostly accumulated on 0 nt from the start codon (Supplementary Fig. 4d, e). This accumulation reflects the fact that the distance from the P-site to the 3′-end of footprints was 19 nt (Supplementary Fig. 4f). Thus, we determined 15 for the A-site offset of 31 nt footprints. Using this as a standard, the A-site offsets of other lengths (27–30, 32 nt) were determined based on the information of which frame was most periodic (Supplementary Fig. 4g). For RNA-Seq, an offset of 15 was used for all mRNA fragments. Reads corresponding to the first and last five codons in CDS were excluded from the calculation. We also omitted reads from the overlapped regions of mitochondrial genes (i.e., *MT-ATP6/8* and *MT-ND4/4L*).

Ribosome footprint changes were calculated with the DESeq2 package^47^. The values were renormalized to the mean of the footprint change of mitochondrial genome-encoded genes, used as the internal spike-in^27^. Ribosome occupancies, which are ribosome profiling counts normalized by RNA-seq counts, were also measured by DESeq2. The significance was calculated by a likelihood ratio test in a generalized linear model. The Gene Ontology analysis was performed with iPAGE v1.2a^52^. All custom scripts used in this study are available upon request. Source codes of softwares for ribosome profiling data analysis were obtained GitHub (https://github.com/ingolia-lab/RiboSeq).

### SFSV NSs in rat primary hippocampal neurons and iPS-derived motor neurons

The hippocampal neurons were dissected from an E18 Slc:Wistar rat, female, according to the standard dissection and dissociation protocol with 0.25% trypsin and DNase I (0.1 mg/ml). Before plating, the cells were electroporated with pI.18-3xFLAG (control), pI.18-SFSVNSs-3xFLAG, or pI.18-SFSVNSs-c1 + 2-Ala-mut-3xFLAG and pCAG-EGFP, together with U6 empty plasmid (NEPA21, Nepa Gene). The hippocampal neurons were then plated on Poly-L-Lysine coated glass coverslips in a 24-well plate, at a density of 100,000 cells/well. The cells were maintained in Neurobasal medium (Thermo Fisher Scientific) containing 2% B27 supplement (Gibco), 1% GlutaMAX, and 1% penicillin/streptomycin. After 2 weeks, Tg (500 nM) was added to the culture medium for either 3 or 24 h. The motor neuron differentiation protocol was described previously^33^. Briefly, human induced-pluripotent stem cells (409B2 from the RIKEN Cell Bank) were seeded in Matrigel-coated plates at 70–80% confluency, in mTeSR Plus medium (STEMCELL Technology) with 10 μM of Y-23632 (Rock inhibitor, Wako). After the cells reached 95% confluency, the neural differentiation was initiated in DMEM/F12 (Sigma) supplemented with 15% knockout serum replacement (Gibco), 1% GlutaMAX (Gibco), 1% non-essential amino acids (Sigma), 10 μM SB431542 (Wako), and 100 nM LDN-193189 (Wako) for the initial 6 days of culture. The cells were treated with 1 μM retinoic acid, 1 μM Smoothened agonist (Wako), 10 μM SU-5402 (Sigma), and 10 μM DAPT (Sigma) from the 4th to 12th days of culture. The motor neurons were then dissociated by Accutase (Innovative Cell Technology) for 20–25 min and replated in Matrigel-coated 24-well plates containing glass coverslips, at a density of 100,000 cells/well. The motor neurons were maintained in Neurobasal medium containing 2% B27 supplement, 1% GlutaMAX, 1% penicillin/streptomycin, and brain-derived neurotrophic factor (20 ng/ml). At 2 days after the cells were replated, the neurons were transfected with pI.18-3xFLAG (control), pI.18-SFSVNSs-3xFLAG, or pI.18-SFSVNSs-c1 + 2-Ala-mut-3xFLAG, together with the pCAG-EGFP plasmid, using Lipofectamine 3000. Three days after the transfection, Tg (500 nM) was added to the culture medium for 2 h. The neurons were fixed by 4% paraformaldehyde for 15 min and permeabilized by 0.1% Triton X-100 for 5 min, followed by blocking with 1% BSA. The cells were then incubated with an anti-ATF4 rabbit antibody (Cell Signaling Technologies, D4B8, 1 : 500), an anti-GFP mouse antibody (DHSB, DSHB-GFP-1D2, 1 : 50), an anti-beta 3 Tubulin mouse antibody (BioLegend, TUJ1, 1 : 1000), and an anti-GADD34 rabbit antibody (Proteintech, 10449-1-AP, 1 : 500) in PBS at 4 °C overnight. On the next day, the cells were further incubated with Alexa Fluor 555-conjugated anti-rabbit IgG (Thermo Fisher Scientific, A27039, 1 : 1000) and Alexa Fluor 647-conjugated anti-mouse IgG (ThermAo Fisher Scientific, A21235 1 : 1000) in PBS at room temperature for 3 h with Hoechst 33342 for nuclear staining, followed by three washes with PBS. The fluorescent images were acquired with an Axio Observer inverted microscope (Zeiss) and a Nikon confocal microscope (A1R).

### ATF4 intensity quantification

The rat primary neurons and motor neurons co-expressing SFSV NSs and GFP (target neurons) were chosen for the ATF4 expression analysis in the confocal and fluorescent images. The images of the ATF4 channel (Red channel) containing target neurons were converted to 16-bit monochrome images, and then the ROI was determined according to the area of the nucleus indicated by Hoechst staining. The mean intensities of the ATF4-staining signals in the ROIs were then calculated. All post-image analyses were conducted by using Fiji (http://imagej.nih.gov/ij/, version 1.53). At least three (Fig. 6c), four (Fig. 6d), and seven (Fig. 5e) neurons were scored for the quantification of ATF4 intensity.

### Sholl analysis

The rat primary hippocampal neurons and motor neurons expressing GFP were examined by a Sholl analysis. The images of neurons were binarized and clarified by removing noise (pixel < 2). After the center of cells (soma) was manually set, the Sholl analysis was performed by using the Sholl analysis plugin in Fiji (https://imagej.net/plugins/sholl-analysis). The parameters of the Sholl analysis are as follows: starting=10 µm ending=200 µm radius_step=5 µm #_samples=1 integration=Mean enclosing cut_off=1 #_primary = [], infer from starting radius, fit profile and compute descriptors, linear with polynomial = [Best fitting degree], most informative and normalized by Area. At least five neurons (Figs. 5d and 6e) were scored. The macro command for the Sholl analysis will be provided upon request.

### Statistics

All of the details for the statistical tests with “*n*” values are indicated in the relevant figure legends and “Methods” sections.

### Reporting summary

Further information on research design is available in the Nature Research Reporting Summary linked to this article.

## Supplementary information

Supplementary Information

Reporting Summary

## Data Availability

The data that support this work are available from the corresponding author upon reasonable request. Structural models used in the study are all available at the Protein Data Bank 3ECS, 5B04, 5OOO, 6O85, 6O9Z, 7D44, 7D46). The cryo-EM maps and the coordinates of the refined models generated for this study are available from the Electron Microscopy Data Bank (EMDB) and PDB under the following accession numbers: eIF2B•SFSV NSs (EMD-32023, PDB 7VLK), eIF2B•SFSV NSs•1-eIF2 (EMD-31474, PDB 7F66), and eIF2B•SFSV NSs•2-eIF2 (EMD-31475, PDB 7F67). The amino acid sequences used for alignment are all available at UniProt Knowledgebase (UniProt: Q14232, H9ETU6, A0A337RW87, F1P8M6, G1SJS2, Q0IIF2, A0A286ZJA8, F6R6B2, A0A286Y0D9, Q64270, Q99LC8, F1NLG8, A0A0R4ILD9, Q9USP0, P14741). The ribosome profiling and RNA-Seq results from this study have been deposited in the National Center for Biotechnology Information (NCBI) under accession code GSE174764. Source data are provided with this paper.

## Source data

Source Data

## Code availability

The codes for deep sequencing data analysis have been deposited in Zenodo (10.5281/zenodo.5675739).
