# Supplementary material for: eIF2B-capturing viral protein NSs suppresses the integrated stress response
Source: Nat Commun. 2021 Dec 7;12:7102. doi: 10.1038/s41467-021-27337-x (PMC8651795; doi:10.1038/s41467-021-27337-x)
Supplement: Supplementary file 1 — Supplementary Information [file 41467_2021_27337_MOESM1_ESM.pdf]

eIF2B-capturing viral protein NSs suppresses the integrated stress response

Kazuhiro Kashiwagi[1,7], Yuichi Shichino[2,7], Tatsuya Osaki[3,7], Ayako Sakamoto[1], Madoka Nishimoto[1], Mari Takahashi[1], Mari Mito[2], Friedemann Weber[4], Yoshiho Ikeuchi[3,5], Shintaro Iwasaki[2,6], & Takuhiro Ito[1]

1. Laboratory for Translation Structural Biology, RIKEN Center for Biosystems Dynamics Research, Tsurumi-ku, Yokohama 230-0045, Japan
2. RNA Systems Biochemistry Laboratory, RIKEN Cluster for Pioneering Research, Wako, Saitama 351-0198, Japan
3. Institute of Industrial Science, The University of Tokyo, Meguro-ku, Tokyo 153-8505, Japan
4. Institute for Virology, FB10-Veterinary Medicine, Justus-Liebig University, Giessen D-35392, Germany
5. Institute for AI and Beyond, The University of Tokyo, Bunkyo-ku, Tokyo 113-8655, Japan
6. Department of Computational Biology and Medical Sciences, Graduate School of Frontier Sciences, The University of Tokyo, Kashiwa, Chiba 277-8561, Japan

7. These authors contributed equally: Kazuhiro Kashiwagi, Yuichi Shichino, Tatsuya Osaki

Correspondence to Yoshiho Ikeuchi (yikeuchi@iis.u-tokyo.ac.jp), Shintaro Iwasaki (shintaro.iwasaki@riken.jp), & Takuhiro Ito (takuhiro.ito@riken.jp)

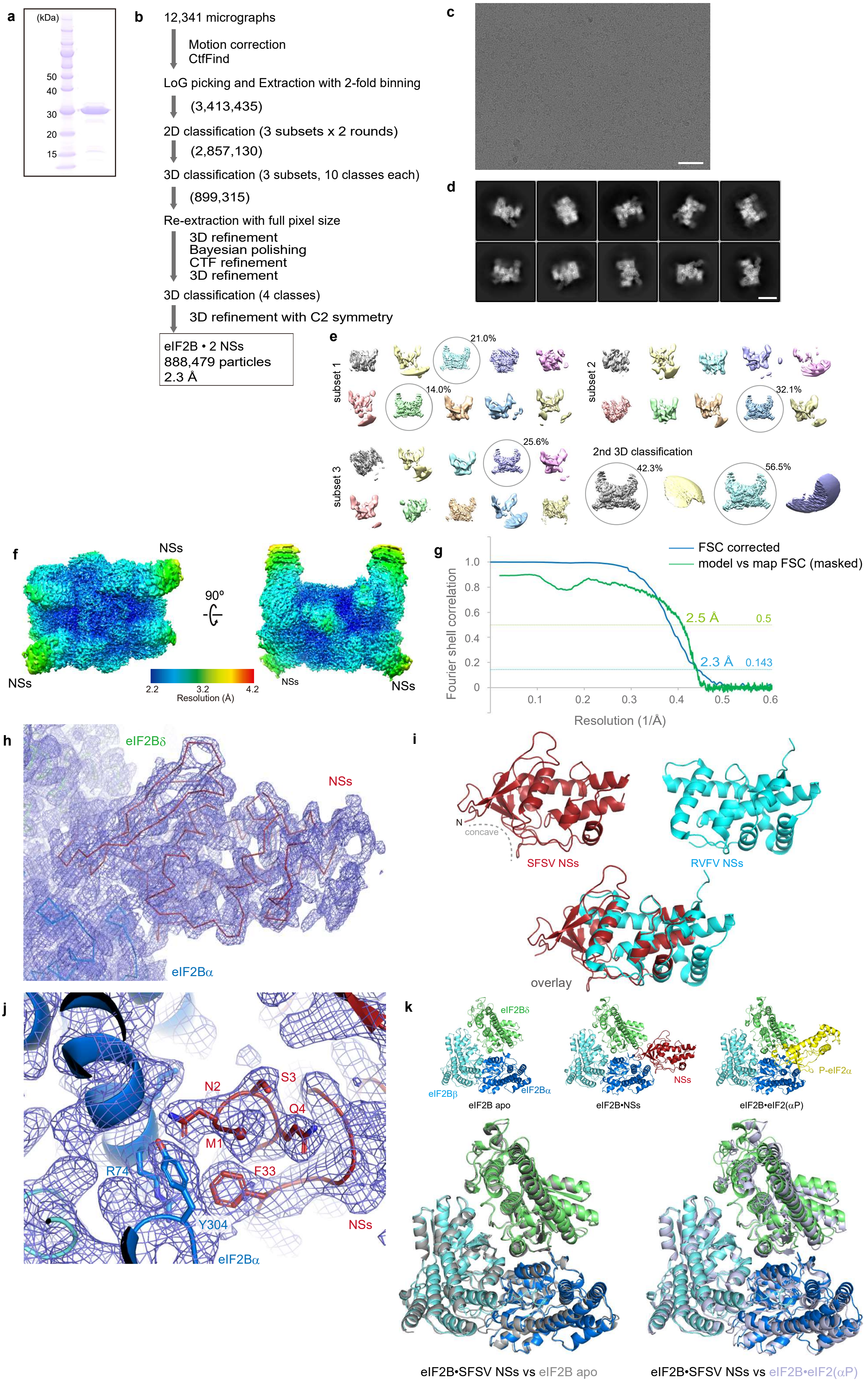

a
(kDa)
50
40
30
20
15
b
12,341 micrographs
Motion correction
CtfFind
LoG picking and Extraction with 2-fold binning
(3,413,435)
2D classification (3 subsets x 2 rounds)
(2,857,130)
3D classification (3 subsets, 10 classes each)
(899,315)
Re-extraction with full pixel size
3D refinement
Bayesian polishing
CTF refinement
3D refinement
3D classification (4 classes)
3D refinement with C2 symmetry
eIF2B • 2 NSs
888,479 particles
2.3 Å
c
d
e
subset 1
21.0%
14.0%
subset 2
32.1%
subset 3
25.6%
2nd 3D classification
42.3%
56.5%
f
NSs
90º
NSs
2.2
3.2
4.2
Resolution (Å)
NSs
NSs
g
FSC corrected
model vs map FSC (masked)
Fourier shell correlation
1.0
0.8
0.6
0.4
0.2
0
2.5 Å
0.5
2.3 Å
0.143
0.1
0.2
0.3
0.4
0.5
0.6
Resolution (1/Å)
h
eIF2Bδ
NSs
eIF2Bα
i
N
concave
SFSV NSs
RVFV NSs
overlay
j
N2
S3
Q4
M1
R74
F33
Y304
NSs
eIF2Bα
k
eIF2Bδ
eIF2Bβ
eIF2Bα
eIF2B apo
NSs
eIF2B•NSs
P-eIF2α
eIF2B•eIF2(αP)
eIF2B•SFSV NSs vs eIF2B apo
eIF2B•SFSV NSs vs eIF2B•eIF2(αP)

Supplementary Fig. 1 | Structural details of the eIF2B•SFSV NSs complex.
a, SDS-PAGE analysis of the SFSV NSs protein sample used for the structural and biochemical analyses. The gel was stained with Coomassie Brilliant Blue. Purification of SFSV NSs was not repeated. **b**, Cryo-EM data processing workflow of image processing. Total particle numbers at each stage are shown in parentheses. **c**, A representative image of collected micrographs. Scale bar = 50 nm. 12,341 images were collected. **d**, Representative images of the selected classes at the 2nd round of 2D classification. Scale bar = 10 nm. 2,857,130 particles from 106 classes were selected. **e**, Selected and discarded classes at each 3D classification step. **f**, Local resolution maps of the eIF2B•SFSV NSs complex from two different views. **g**, Fourier shell correlation (FSC) curves of the cryo-EM map. The blue line shows the FSC curve for the 3D reconstruction, and the green line shows the FSC curve calculated between the refined model and the cryo-EM map. **h**, EM density map around SFSV NSs. Some unassigned density is also observed in the distal area from the body of eIF2B. **i**, Structural comparison of SFSV NSs (maroon) and RVFV NSs (cyan, PDB: 5OOO)[21]. **j**, EM density map around the N-terminus of SFSV NSs. The eIF2Bα subunit accommodates the very N-terminus of SFSV NSs. The sidechain of M1 was not resolved, and therefore its Cα position is shown as a sphere. **k**, Comparisons of the conformations of eIF2B regulatory subunits in the eIF2B•SFSV NSs complex, eIF2B apo (PDB: 7D46), and the eIF2B•eIF2(αP) complex structures (PDB: 7D44)[14]. In the lower panels, the subunits of eIF2B are aligned with their eIF2Bβ subunit C-terminal domains. *Lower left*: Comparison of eIF2B•SFSV NSs (color-coded) and eIF2B apo (grey). *Lower right*: Comparison of eIF2B•SFSV NSs (color-coded) and eIF2B•eIF2(αP) (pale blue).
Source data are provided as a Source Data file.

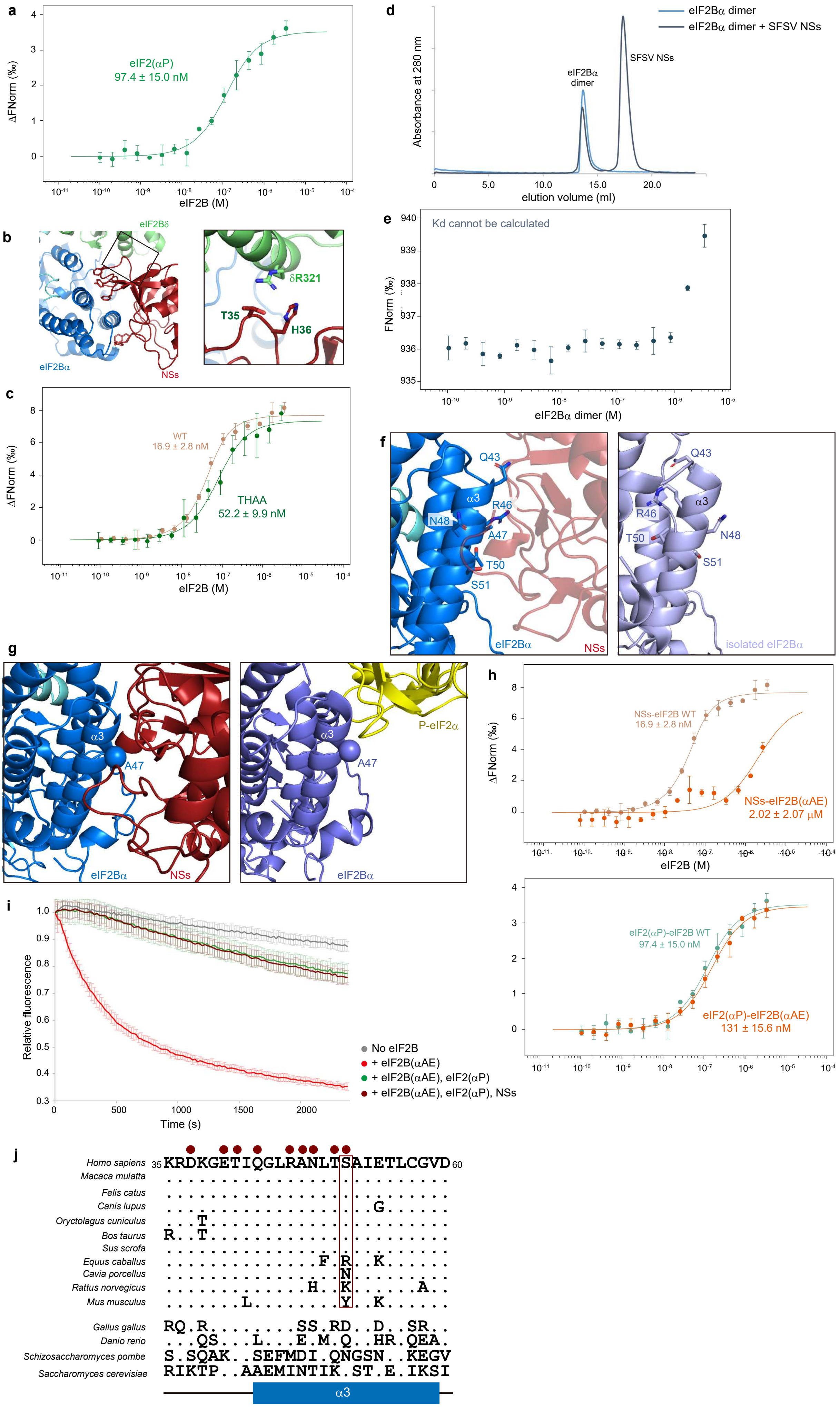

a
eIF2(αP)
97.4 ± 15.0 nM
ΔFNorm (‰)
eIF2B (M)
b
eIF2Bδ
eIF2Bα
NSs
δR321
T35
H36
c
WT
16.9 ± 2.8 nM
THAA
52.2 ± 9.9 nM
ΔFNorm (‰)
eIF2B (M)
d
eIF2Bα dimer
eIF2Bα dimer + SFSV NSs
SFSV NSs
eIF2Bα dimer
Absorbance at 280 nm
elution volume (ml)
e
Kd cannot be calculated
FNorm (‰)
eIF2Bα dimer (M)
f
Q43
α3
R46
N48
A47
T50
S51
eIF2Bα
NSs
isolated eIF2Bα
g
α3
A47
eIF2Bα
NSs
P-eIF2α
h
NSs-eIF2B WT
16.9 ± 2.8 nM
NSs-eIF2B(αAE)
2.02 ± 2.07 μM
ΔFNorm (‰)
eIF2B (M)
eIF2(αP)-eIF2B WT
97.4 ± 15.0 nM
eIF2(αP)-eIF2B(αAE)
131 ± 15.6 nM
i
Relative fluorescence
Time (s)
No eIF2B
+ eIF2B(αAE)
+ eIF2B(αAE), eIF2(αP)
+ eIF2B(αAE), eIF2(αP), NSs
j
Homo sapiens 35 KRDKGETIQGLRANLTSAIETLCGVD 60
Macaca mulatta
Felis catus
Canis lupus . . . . G . . . .
Oryctolagus cuniculus . . T . . . .
Bos taurus R . T . . . .
Sus scrofa
Equus caballus . . . . F . R . . K . . . . . .
Cavia porcellus . . . . N . . . .
Rattus norvegicus . . . . H . . K . . . . . A . .
Mus musculus . . . . L . . . . Y . . K . . . . . .
Gallus gallus RQ.R.......SS.RD..D..SR..
Danio rerio ...QS...L...E.M.Q..HR.QEA.
Schizosaccharomyces pombe S.SQAK..SEFMDI.QNGSN..KEGV
Saccharomyces cerevisiae RIKTP..AAEMINTIK.ST.E.IKSI
α3

Supplementary Fig. 2 | Comparison of the eIF2B-SFSV NSs and eIF2B-eIF2(αP) interactions.
a, MST analysis between eIF2B and eIF2(αP). (n = 3). **b**, The interface between SFSV NSs and the eIF2Bδ subunit. The interacting residues are shown in stick models. **c**, MST analysis between eIF2B and SFSV NSs T35A, H36A mutant (THAA; green, n = 3). The control (wild type SFSV NSs) shown in beige was the same experiment as depicted by the maroon line in **Fig. 2a**. **d**, **e**, The analysis of the interaction between SFSV NSs and the isolated eIF2Bα dimer by size exclusion chromatography (**d**) and MST (**e**). The dissociation constant could not be calculated from the measurements (n = 3). **f**, Comparison of the helix α3 of eIF2Bα in the eIF2B•SFSV NSs complex (*left*) and the isolated eIF2B dimer (*right*; PDB: 3ECS)[24]. Some residues involved in the interaction with SFSV NSs are shown in stick models. **g**, Comparison of the interaction of helix α3 of eIF2Bα with SFSV NSs (*left*) and that with eIF2(αP) (*right*; PDB: 7D44)[14]. A47 of eIF2Bα (shown as spheres) resides at the interface for SFSV NSs, but it is not involved in the interaction with eIF2(αP). **h**, MST analysis between SFSV NSs and eIF2B(αA47E) mutant [eIF2B(αAE); orange] (*upper*), and analysis between eIF2(αP) and eIF2B(αAE) (*lower*) (n = 3). Controls (wild type eIF2B) (the beige line in the upper panel and the green line in the lower panel) were the same experiments as in **Fig. 2a** (maroon line) and in (**a**) of this figure (green line), respectively. **i**, Assay of guanine nucleotide exchange activity of eIF2B(αA47E), the inhibition by eIF2(αP), and the suppression by SFSV NSs. The concentrations of the factors are the same as in **Fig. 2d**. [n = 4 for eIF2+ eIF2B(αA47E) (red), and n = 5 for the rest of the experiments]. **j**, Multiple alignment of amino acid sequences around the helix α3 of eIF2Bα from mammals and non-mammalian eukaryotes [*Homo sapiens* (UniProt: Q14232), *Macaca mulatta* (H9ETU6), *Felis catus* (A0A337RW87), *Canis lupus familiaris* (F1P8M6), *Oryctolagus cuniculus* (G1SJS2), *Bos taurus* (Q0IIF2), *Sus scrofa* (A0A286ZJA8), *Equus caballus* (F6R6B2), *Cavia porcellus* (A0A286Y0D9), *Rattus norvegicus* (Q64270), *Mus musculus* (Q99LC8), *Gallus gallus* (F1NLG8), *Danio rerio* (A0A0R4ILD9), *Schizosaccharomyces pombe* (Q9USP0) and *Saccharomyces cerevisiae* (P14741)]. SFSV NSs-interacting residues are marked with maroon circles and the variable S51 is highlighted.
a, **c**, **e**, **h**, and **i**: data are presented as mean values +/- standard deviations at each concentration or time point, and n means the number of independent experiments. Source data are provided as a Source Data file

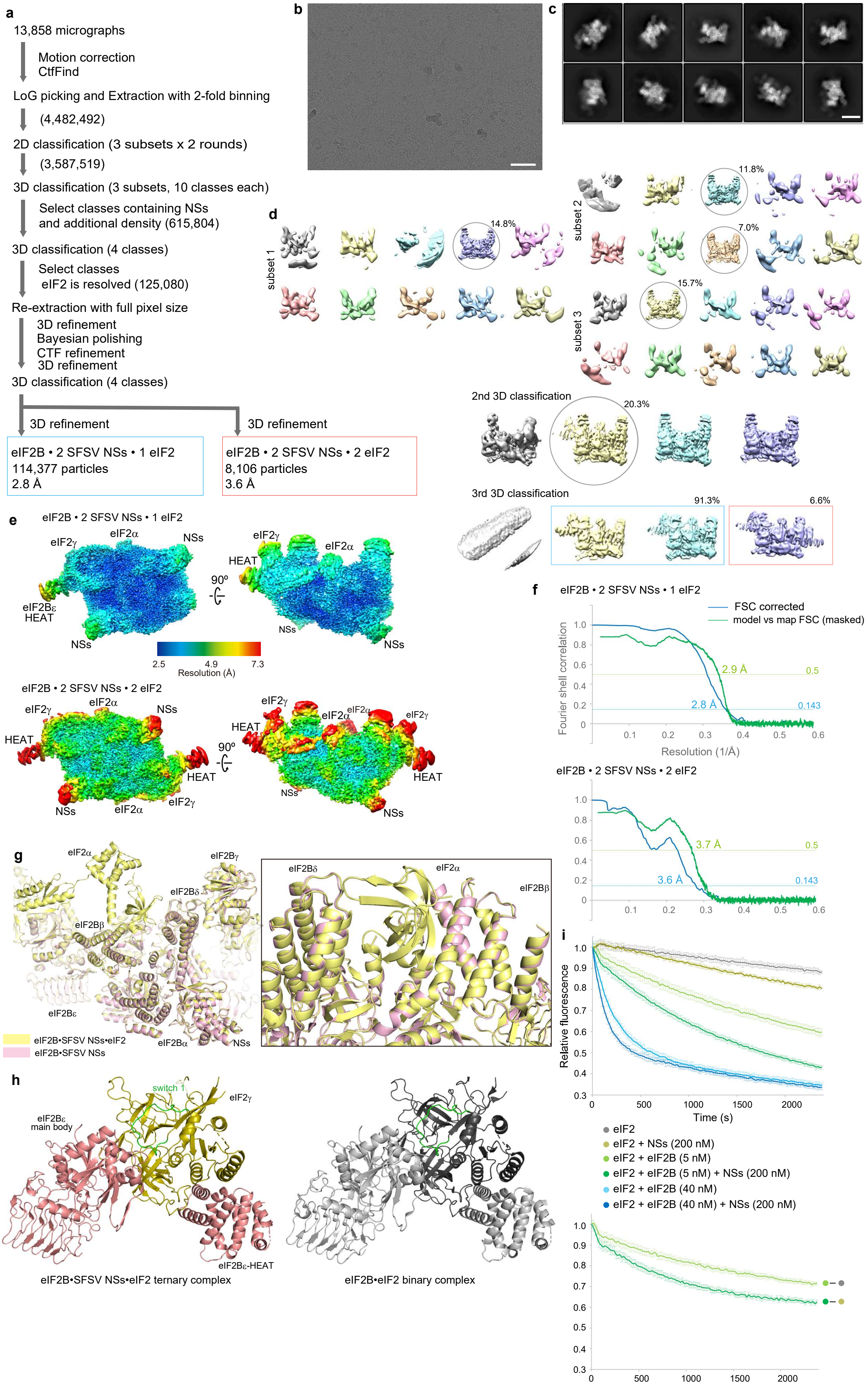
a
13,858 micrographs
Motion correction
CtfFind
LoG picking and Extraction with 2-fold binning
(4,482,492)
2D classification (3 subsets x 2 rounds)
(3,587,519)
3D classification (3 subsets, 10 classes each)
Select classes containing NSs
and additional density (615,804)
3D classification (4 classes)
Select classes
eIF2 is resolved (125,080)
Re-extraction with full pixel size
3D refinement
Bayesian polishing
CTF refinement
3D refinement
3D classification (4 classes)
3D refinement
3D refinement
eIF2B • 2 SFSV NSs • 1 eIF2
114,377 particles
2.8 Å
eIF2B • 2 SFSV NSs • 2 eIF2
8,106 particles
3.6 Å
b
c
d
subset 1
14.8%
subset 2
11.8%
7.0%
subset 3
15.7%
2nd 3D classification
20.3%
3rd 3D classification
91.3%
6.6%
e
eIF2B • 2 SFSV NSs • 1 eIF2
eIF2γ
eIF2α
NSs
eIF2Bε
HEAT
NSs
HEAT
90º
2.5
4.9
7.3
Resolution (Å)
eIF2B • 2 SFSV NSs • 2 eIF2
HEAT
f
eIF2B • 2 SFSV NSs • 1 eIF2
FSC corrected
model vs map FSC (masked)
Fourier shell correlation
2.9 Å
0.5
2.8 Å
0.143
Resolution (1/Å)
eIF2B • 2 SFSV NSs • 2 eIF2
3.7 Å
3.6 Å
g
eIF2Bγ
eIF2Bδ
eIF2Bβ
eIF2Bε
eIF2Bα
eIF2B•SFSV NSs•eIF2
eIF2B•SFSV NSs
h
switch 1
eIF2Bε
main body
eIF2Bε-HEAT
eIF2B•SFSV NSs•eIF2 ternary complex
eIF2B•eIF2 binary complex
i
Relative fluorescence
Time (s)
eIF2
eIF2 + NSs (200 nM)
eIF2 + eIF2B (5 nM)
eIF2 + eIF2B (5 nM) + NSs (200 nM)
eIF2 + eIF2B (40 nM)
eIF2 + eIF2B (40 nM) + NSs (200 nM)

Supplementary Fig. 3 | Cryo-EM data processing and structural details of the eIF2B•SFSV NSs•unphosphorylated eIF2 ternary complex.

a, Workflow of image processing. Total particle numbers at each stage are shown in parentheses. **b**, A representative image of collected micrographs. Scale bar = 50 nm. 13,858 images were collected. **c**, Representative images of the selected classes at the 2nd round of 2D classification. Scale bar = 10 nm. 3,587,519 particles from 62 classes were selected. **d**, Selected and discarded classes at each 3D classification step. **e**, Local resolution maps of the eIF2B•SFSV NSs•unphosphorylated eIF2 complexes from two different views. **f**, Fourier shell correlation (FSC) curves of the cryo-EM maps. The blue lines show the FSC curves for the 3D reconstructions, and the green lines show the FSC curves calculated between the refined models and the cryo-EM maps. **g**, Structural comparison of the eIF2B•SFSV NSs complex (pink) and the eIF2B•SFSV NSs•unphosphorylated eIF2 ternary complex (yellow), and close-up view of the interface for the unphosphorylated eIF2 (right panel). Structures were aligned with their eIF2Bβ subunit C-terminal domains. **h**, Comparison of the interactions between the eIF2Bε subunit and the eIF2γ subunit in the eIF2B•SFSV NSs•unphosphorylated eIF2 ternary complex (pink and olive) and the eIF2B•eIF2 binary complex structures (grey and black; PDB: 6O85)[3]. The switch 1 region of eIF2γ is shown in green. **i**, Guanine nucleotide exchange activity of eIF2B in the presence and absence of SFSV NSs. Data are presented as mean values +/- standard deviations at each time point [n = 4 for eIF2+SFSV NSs (200 nM) (olive), and n = 5 for the rest of the experiments. n means the number of independent experiments]. When SFSV NSs was included in the measurement solution, a small increase of eIF2B-independent dissociation of GDP was observed (compare grey and olive lines). In the lower graph, this effect is subtracted from the eIF2+eIF2B (5 nM) (light green) and eIF2+eIF2B (5 nM)+SFSV NSs (200 nM) measurements (green).

Source data are provided as a Source Data file.

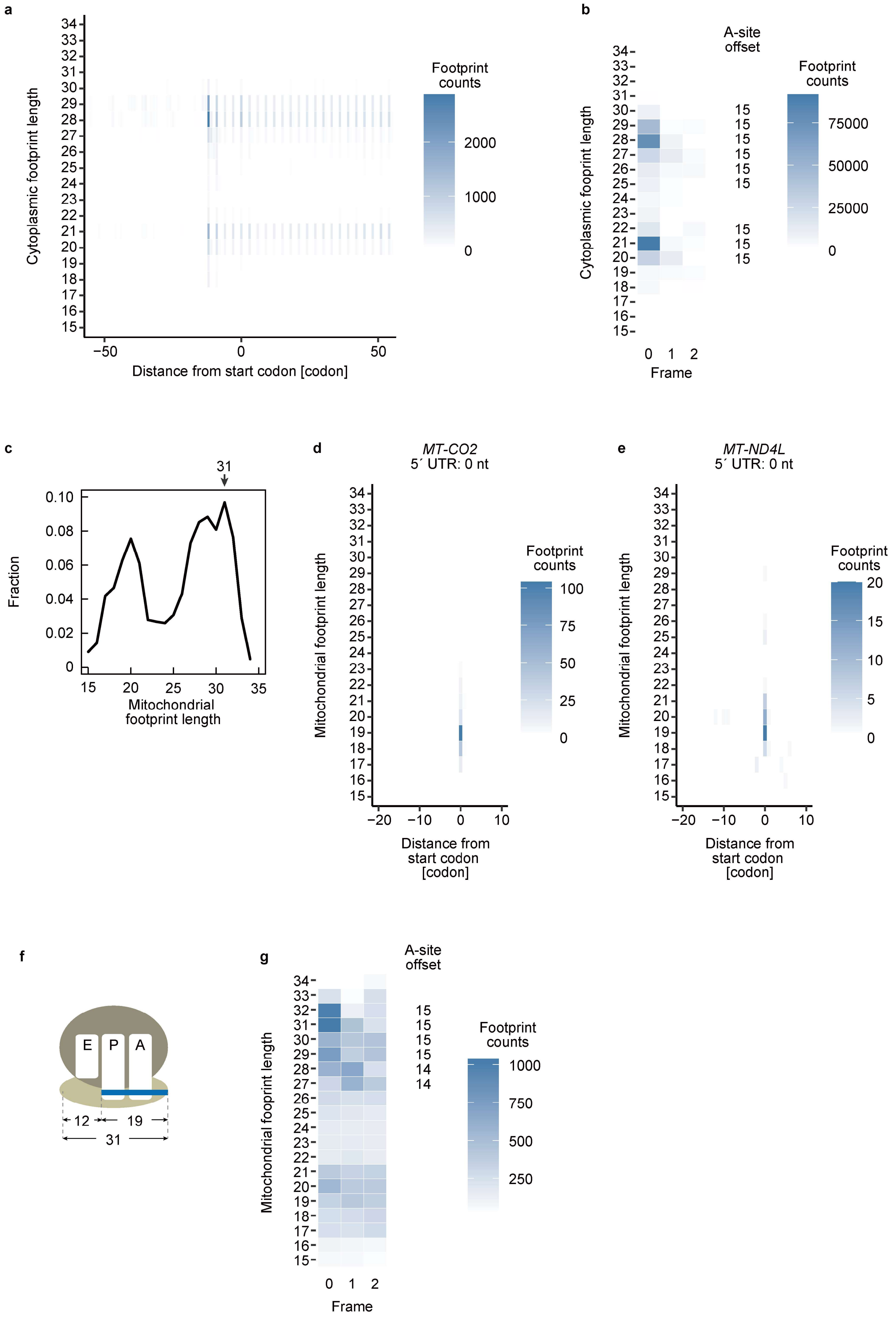

a
Cytoplasmic footprint length
Distance from start codon [codon]
Footprint counts
b
A-site offset
Cytoplasmic fooprint length
Frame
Footprint counts
c
31
Fraction
Mitochondrial footprint length
d
MT-CO2
5´ UTR: 0 nt
Mitochondrial footprint length
Distance from start codon [codon]
Footprint counts
e
MT-ND4L
5´ UTR: 0 nt
Mitochondrial footprint length
Distance from start codon [codon]
Footprint counts
f
E
P
A
12
19
31
g
A-site offset
Mitochondrial fooprint length
Frame
Footprint counts

Supplementary Fig. 4 | Metagene analysis for cytoplasmic and mitochondrial footprints in vector-transfected control sample treated with 0 nM Tg, replicate 1.

a, 2D-metagene plots for the 5′ end of cytoplasmic footprints. The x-axis indicates relative position from the start codon and the y-axis indicates footprint length. **b**, Tile plot of corresponding reading frames for the 5′ end of cytoplasmic footprints. A-site offsets determined in **a** and **b** are shown in the middle. **c**, Distribution of mitochondrial footprint lengths. **d** and **e**, Tile plots for the 5′ end of mitochondrial footprints on *MT-CO2* (**d**) and *MT-ND4L* (**e**) around the start codons. **f**, Schematic of the mitochondrial footprint for the ribosome on the start codon of the leaderless transcripts. **g**, Tile plot of corresponding reading frames for the 5′ end of mitochondrial footprints. A-site offsets determined in **c-g** are shown in the middle.

a, **b**, **d**, **e**, and **g**: the footprint count scales are shown at the color bars.

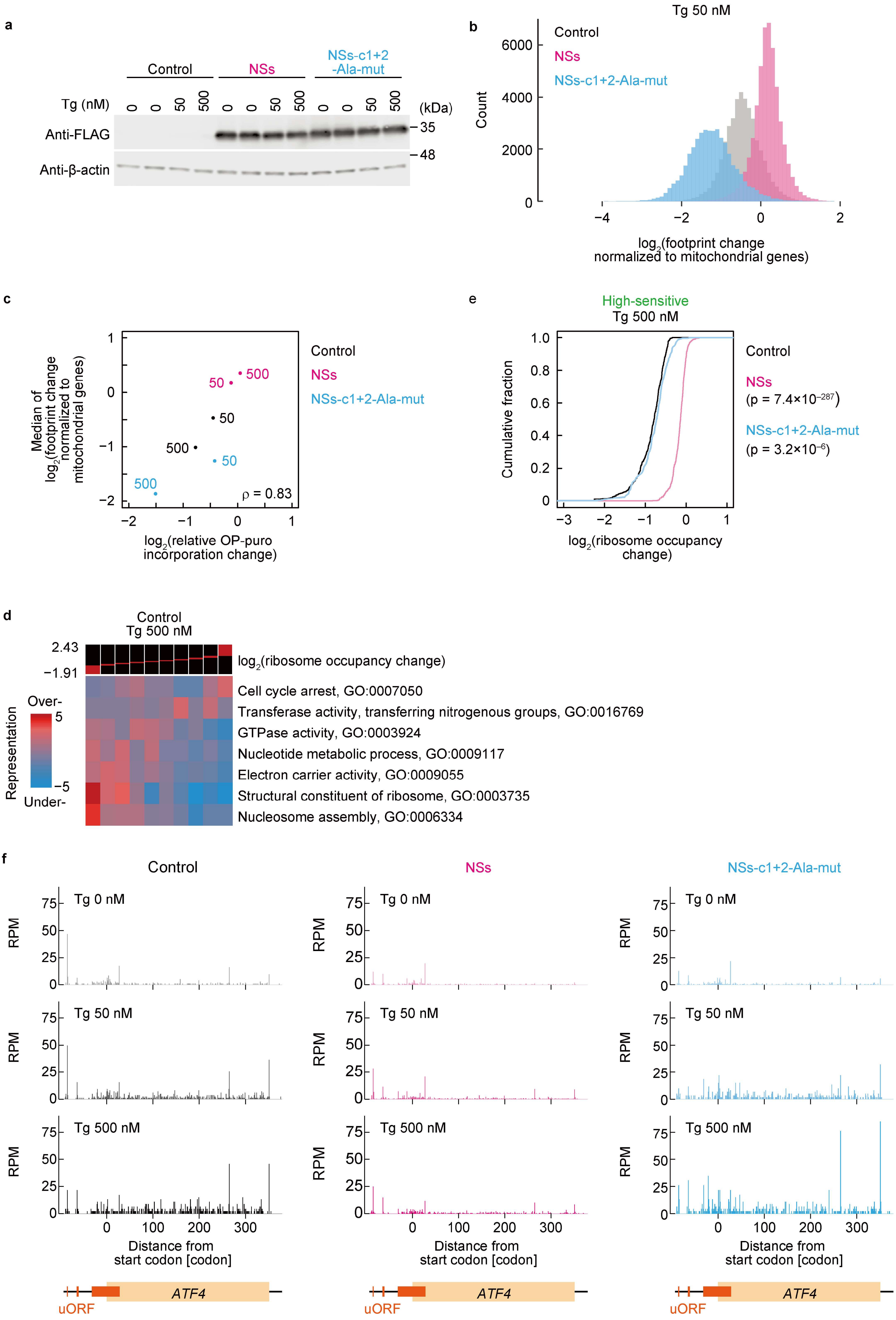
a
Control
NSs
NSs-c1+2
-Ala-mut
Tg (nM)
0 0 50 500 0 0 50 500 0 0 50 500
(kDa)
Anti-FLAG
35
48
Anti-β-actin
b
Tg 50 nM
Control
NSs
NSs-c1+2-Ala-mut
Count
6000
4000
2000
0
−4 −2 0 2
log2(footprint change
normalized to mitochondrial genes)
c
Median of
log2(footprint change
normalized to
mitochondrial genes)
1
0
−1
−2
500
50
50
500
50
500
ρ = 0.83
−2 −1 0 1
log2(relative OP-puro
incorporation change)
Control
NSs
NSs-c1+2-Ala-mut
e
High-sensitive
Tg 500 nM
Cumulative fraction
1.0
0.8
0.6
0.4
0.2
0
−3 −2 −1 0 1
log2(ribosome occupancy
change)
Control
NSs
(p = 7.4×10−287)
NSs-c1+2-Ala-mut
(p = 3.2×10−6)
d
Control
Tg 500 nM
2.43
−1.91
log2(ribosome occupancy change)
Over-
5
Representation
−5
Under-
Cell cycle arrest, GO:0007050
Transferase activity, transferring nitrogenous groups, GO:0016769
GTPase activity, GO:0003924
Nucleotide metabolic process, GO:0009117
Electron carrier activity, GO:0009055
Structural constituent of ribosome, GO:0003735
Nucleosome assembly, GO:0006334
f
Control
NSs
NSs-c1+2-Ala-mut
Tg 0 nM
Tg 50 nM
Tg 500 nM
RPM
75
50
25
0
0 100 200 300
Distance from
start codon [codon]
uORF
ATF4

g

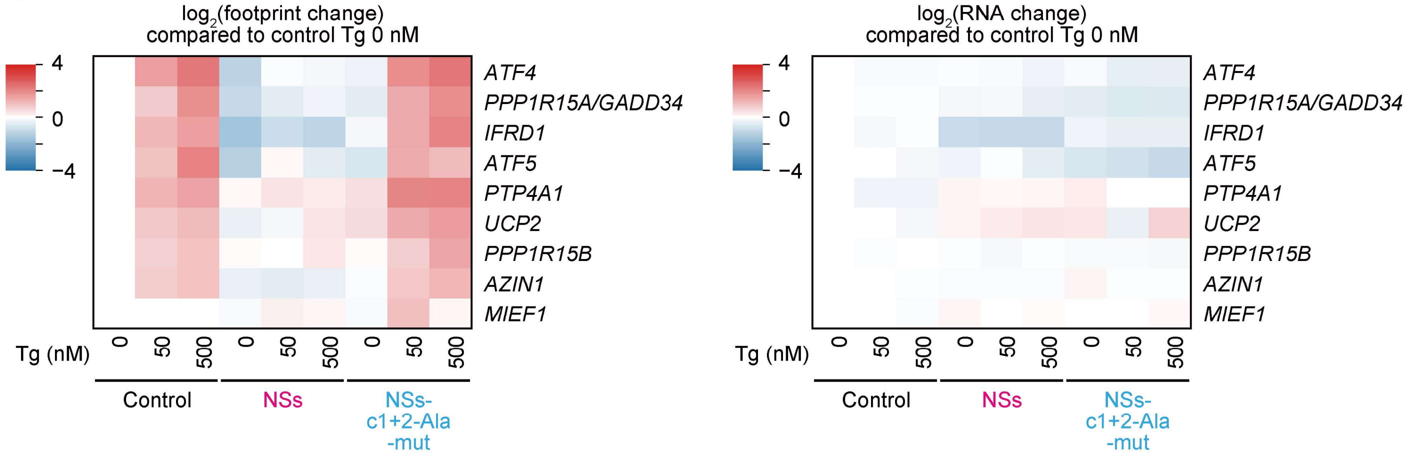
log2(footprint change)
compared to control Tg 0 nM
4
0
−4
ATF4
PPP1R15A/GADD34
IFRD1
ATF5
PTP4A1
UCP2
PPP1R15B
AZIN1
MIEF1
Tg (nM)
0
50
500
0
50
500
0
50
500
Control
NSs
NSs-c1+2-Ala-mut
log2(RNA change)
compared to control Tg 0 nM
4
0
−4
ATF4
PPP1R15A/GADD34
IFRD1
ATF5
PTP4A1
UCP2
PPP1R15B
AZIN1
MIEF1
Tg (nM)
0
50
500
0
50
500
0
50
500
Control
NSs
NSs-c1+2-Ala-mut

Supplementary Fig. 5 | SFSV NSs counteracts the translation repression upon thapsigargin treatment, as revealed by ribosome profiling.

a, Western blot for FLAG-tagged SFSV NSs or SFSV NSs-c1+2-Ala-mut in Tg-treated cells, with β-actin as a loading control. Two replicates for Tg 0 nM and a single replicate for Tg 50 or 500 nM were shown. **b**, Histogram of the number of transcripts along the footprint changes in cells treated with 50 nM Tg. Data were normalized to the mean of the footprint changes of mitochondrial genome-encoded genes (used as internal spike-ins). Bin width is 0.1. **c**, Comparison between relative OP-puro incorporation change (Fig. 4a, b) and median of footprint change normalized to mitochondrial ribosome footprints (Fig. 4c and Supplementary Fig. 5b). The concentrations of Tg (nM) are shown next to the points. ρ, Spearman's rank correlation coefficient. **d**, GO analysis of ribosome occupancy change in control vector-transfected cells treated with 500 nM Tg and visualized by iPAGE (ref. 52). The scale of log-transformed hypergeometric p value is shown at the color bars. **e**, Cumulative distribution of high-sensitive mRNAs (defined in Fig. 4d) along ribosome occupancy changes in control, SFSV NSs-expressing, or SFSV NSs-c1+2-Ala-mut-expressing cells treated with 500 nM Tg. The p-value was calculated by the two-sided Mann-Whitney *U* test. **f**, Read distributions along *ATF4* mRNA in control, SFSV NSs-expressing, or SFSV NSs-c1+2-Ala-mut-expressing cells treated with Tg. The A-site positions of the footprints are shown. RPM: Reads per million reads. **g**, Heatmap of footprint and RNA changes on uORF-containing stress-resistant mRNAs (identified in ref. 28), compared to control vector-transfected cells treated with 0 nM Tg. The $\log_2$-fold change scale is shown at the color bars.

Source data are provided as a Source Data file.

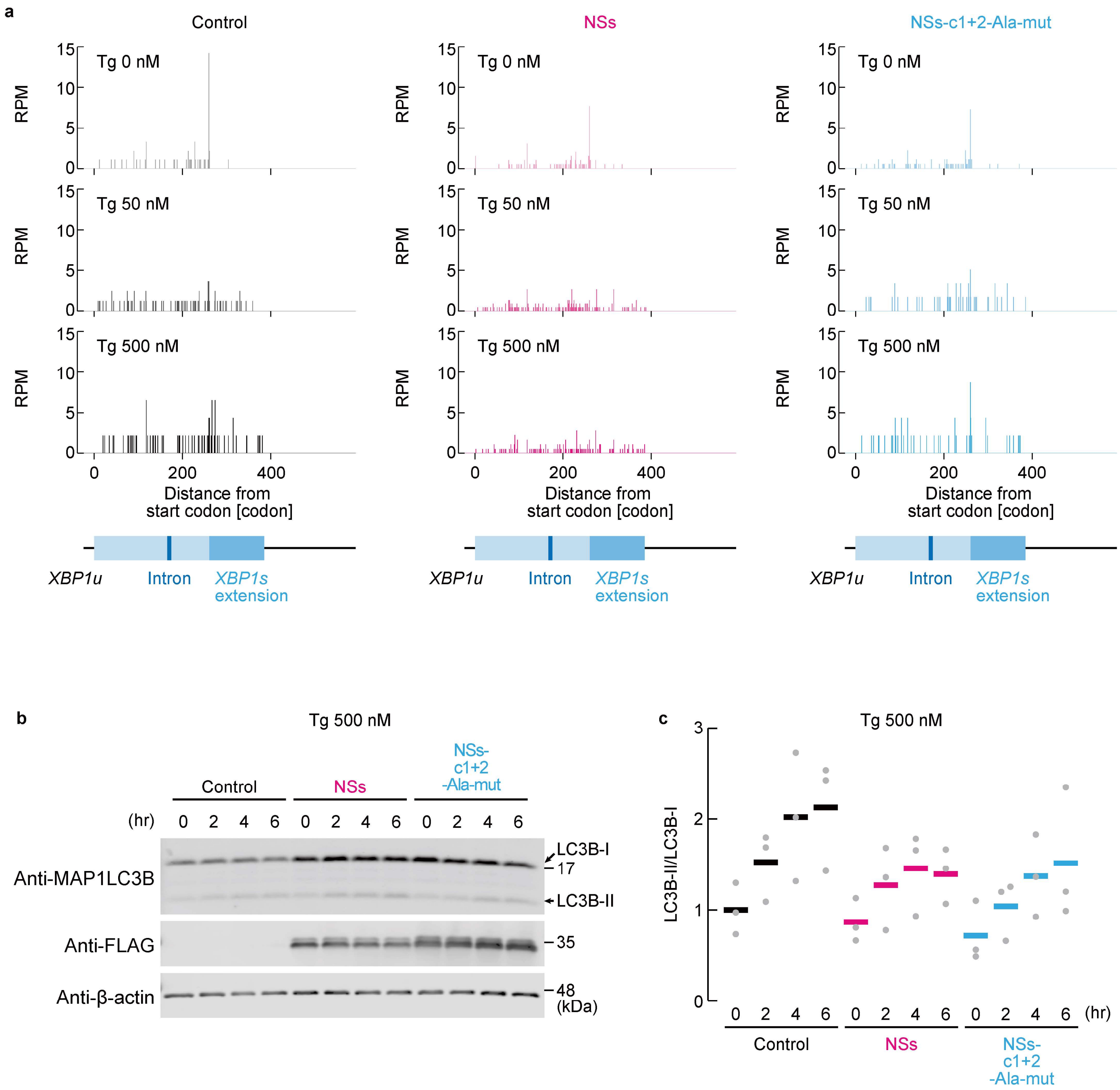
a
Control
NSs
NSs-c1+2-Ala-mut
Tg 0 nM
Tg 50 nM
Tg 500 nM
RPM
Distance from start codon [codon]
XBP1u
Intron
XBP1s extension
b
Tg 500 nM
Control
NSs
NSs-c1+2-Ala-mut
(hr) 0 2 4 6 0 2 4 6 0 2 4 6
Anti-MAP1LC3B
Anti-FLAG
Anti-β-actin
LC3B-I
LC3B-II
17
35
48
(kDa)
c
Tg 500 nM
LC3B-II/LC3B-I
0 2 4 6 0 2 4 6 0 2 4 6 (hr)
Control
NSs
NSs-c1+2-Ala-mut

Supplementary Fig. 6 | SFSV NSs does not affect the IRE1 branch and autophagy induction upon the ER stress response.

a, Read distributions along *XBP1u* mRNA in control, SFSV NSs-expressing, or SFSV NSs-c1+2-Ala-mut-expressing cells treated with Tg. The A-site positions of the footprints are shown. RPM: Reads per million reads. **b**, Representative Western blot image for LC3B in control, SFSV NSs-expressing, or SFSV NSs-c1+2-Ala-mut-expressing cells. Cells were also treated with 500 nM Tg for the indicated incubation times, and β-actin was used as a loading control. Representative images of three biologically independent replicates are shown. **c**, Quantification of the ratio between the LC3B-I and LC3B-II levels in **b**. Data of three replicates (points) and the means (bars) are shown.

Source data are provided as a Source Data file.

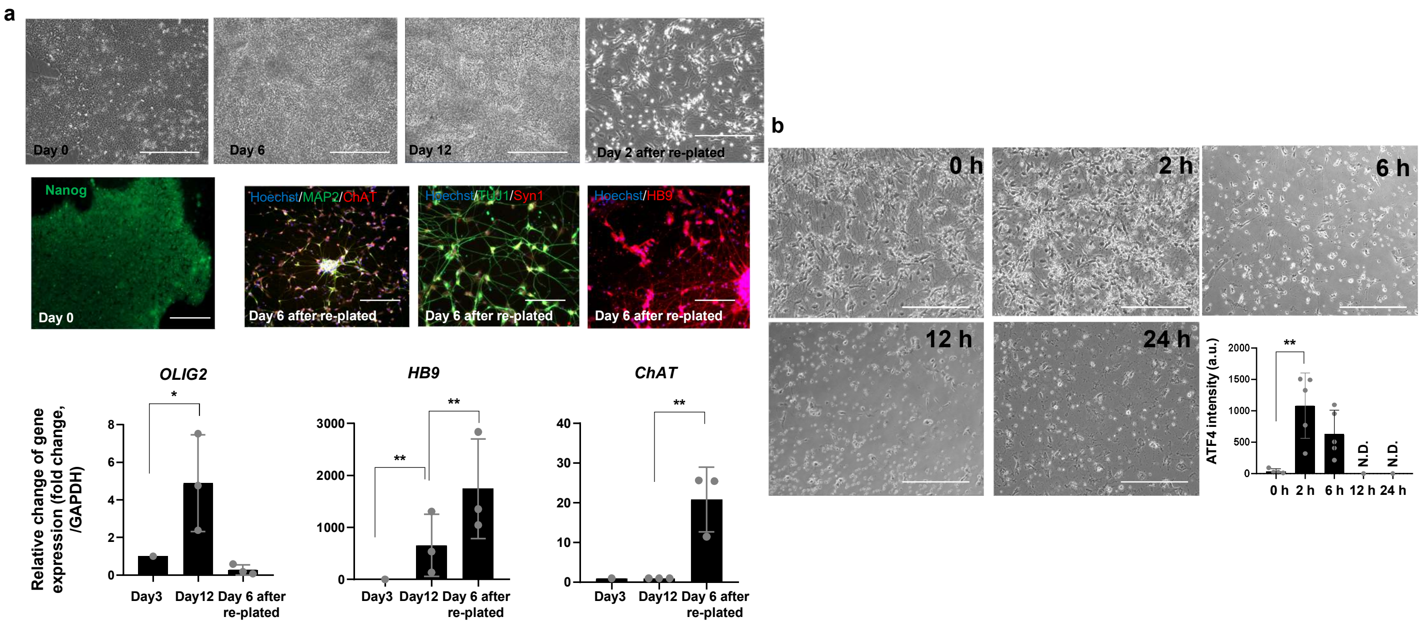
a
Day 0
Day 6
Day 12
Day 2 after re-plated
Nanog
Day 0
Hoechst/MAP2/ChAT
Day 6 after re-plated
Hoechst/TUJ1/Syn1
Day 6 after re-plated
Hoechst/HB9
Day 6 after re-plated
OLIG2
HB9
ChAT
Relative change of gene expression (fold change, /GAPDH)
Day3
Day12
Day 6 after re-plated
b
0 h
2 h
6 h
12 h
24 h
ATF4 intensity (a.u.)
N.D.
N.D.
0 h 2 h 6 h 12 h 24 h

Supplementary Fig. 7 | Characterization of iPS-derived motor neurons and ISR induction by thapsigargin.

a, Time course of the differentiation into motor neurons from iPS cells. Nanog is the marker for undifferentiated pluripotent stem cells. Immunostaining revealed robust expression of HB9 and ChAT in the differentiated motor neurons. RT-PCRs of OLIG2, HB9, and ChAT support their developmental maturation steps toward motor neurons (n =3). Scale bar = 250 µm; *, $p < 0.05$; **, $p < 0.01$. **b**, Cell survival in multiple treatment durations (0, 2, 6, 12, 24 h) with thapsigargin (500 nM). Quantified results of ATF4 intensity in the nucleus. Not determined (N.D.) in the 12 and 24 h-treatments. Scale bar = 250 µm, at least 4 neurons were scored. n =4, n means the number of independent experiments. *, $p < 0.05$; **, $p < 0.01$; a.u., arbitrary units.

a and **b**: One-way ANOVA with Tukey's multiple comparisons test. Error bars ± SD. Source data are provided as a Source Data file.

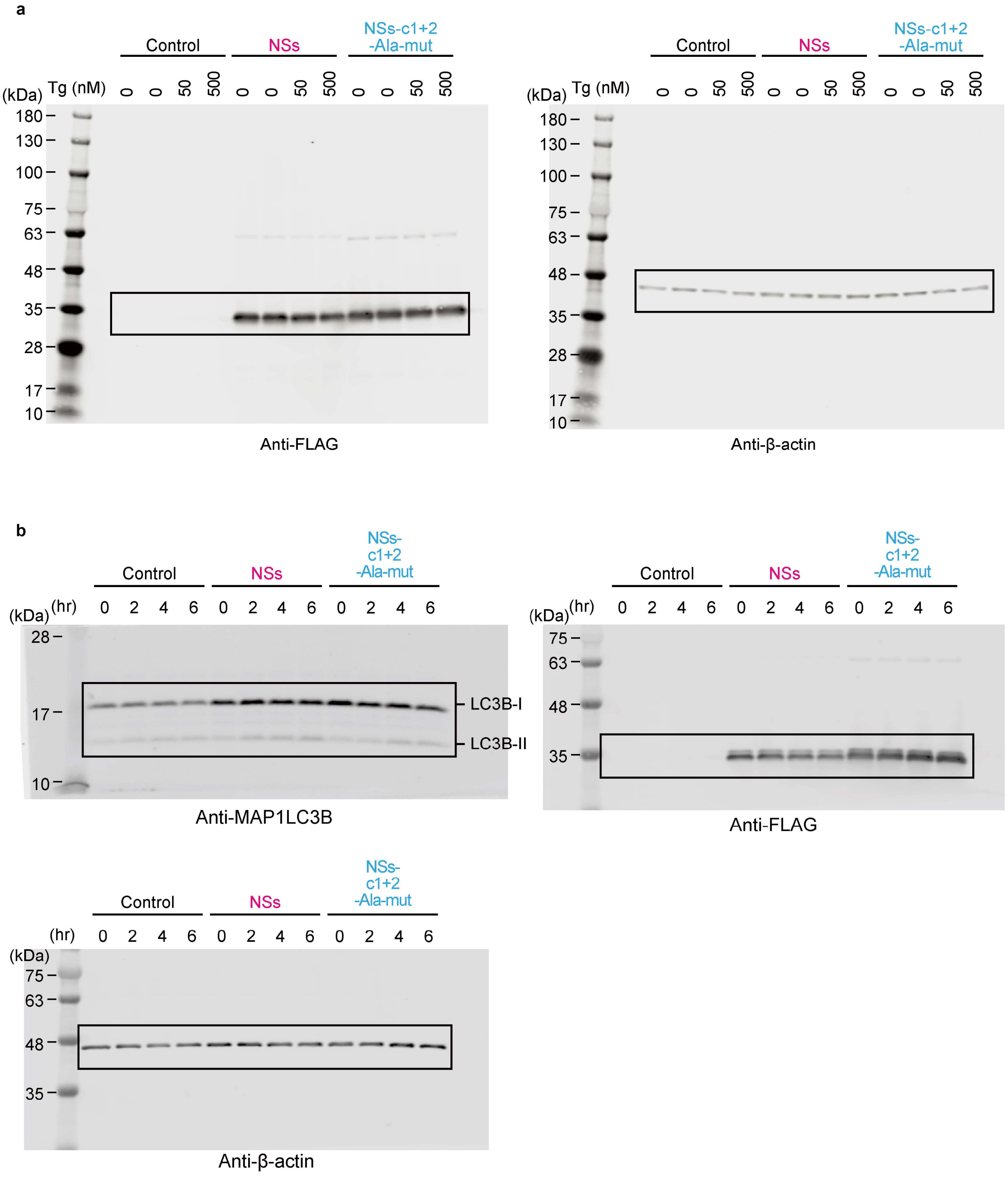
a
Control
NSs
NSs-c1+2
-Ala-mut
(kDa)
Tg (nM)
0
0
50
500
180
130
100
75
63
48
35
28
17
10
Anti-FLAG
Anti-β-actin
b
NSs-
c1+2
-Ala-mut
(hr)
0
2
4
6
LC3B-I
LC3B-II
Anti-MAP1LC3B
Anti-FLAG
Anti-β-actin

Supplementary Fig. 8 | Uncropped images for Western blot.
a and **b**, Uncropped images for Supplementary Fig. 5a (**a**) and 6b (**b**).

Supplementary Table 1 | Cryo-EM data collection, refinement and validation statistics.

	eIF2B•SFSV NSs (EMDB-32023) (PDB: 7VLK)	eIF2B•SFSV NSs•1-eIF2 (EMDB-31474) (PDB: 7F66)	eIF2B•SFSV NSs•2-eIF2 (EMDB-31475) (PDB: 7F67)
**Data collection and processing**			
Magnification	105,000	105,000	
Voltage (kV)	300	300	
Electron exposure ($e^{-}/Å^2$)	50	50	
Defocus range (μm)	−1.0 to −3.0	−1.5 to −3.0	
Pixel size (Å)	0.829	0.829	
Symmetry imposed	C2	C1	
Initial particle images (no.)	3,413,435	4,482,492	
Final particle images (no.)	888,479	114,377	8,106
Map resolution (Å)	2.27	2.76	3.59
FSC threshold	0.143	0.143	0.143
**Refinement**			
Initial model used (PDB code)	6O9Z	6O9Z, 6O85	6O9Z, 6O85
Model resolution (Å)	2.5	2.9	3.7
FSC threshold	0.5	0.5	0.5
Map sharpening B factor ($Å^2$)	−61.0	−56.1	−56.4
Model composition			
Non-hydrogen atoms	28774	33710	37714
Protein residues	3698	4545	5358
B factors ($Å^2$)			
Protein	76.24	101.76	152.71
R.m.s. deviations			
Bond lengths (Å)	0.002	0.003	0.004
Bond angles (°)	0.505	0.583	0.610
Validation			
MolProbity score	1.66	1.68	1.89
Clashscore	6.30	6.44	9.06
Poor rotamers (%)	2.51	0.03	0.09
Ramachandran plot			
Favored (%)	97.97	95.29	94.01
Allowed (%)	2.03	4.67	5.99
Disallowed (%)	0.00	0.04	0.00
*CC*mask	0.84	0.88	0.84
MolProbity score percentile	90th	90th	86th

Supplementary Table 2 | Read counts for ribosome profiling.

	Tg	After filtering and trimming	After non-coding RNA removal	After mapping to genome	After duplicate removal
Control	0 nM #1	50,025,938	4,276,510	4,083,231	3,398,612
	0 nM #2	16,946,552	1,202,894	1,149,456	924,891
	50 nM	23,893,610	1,131,201	1,004,186	836,661
	500 nM	25,039,753	688,271	567,688	470,694
NSs	0 nM #1	20,058,029	1,933,338	1,851,894	1,507,450
	0 nM #2	24,844,148	2,544,335	2,425,935	1,987,417
	50 nM	37,285,699	2,948,554	2,742,785	2,313,054
	500 nM	30,562,385	2,329,220	2,165,104	1,825,287
NSs-c1+c2-Ala-mut	0 nM #1	22,509,524	2,419,933	2,334,862	1,905,539
	0 nM #2	20,103,302	2,402,371	2,199,133	1,799,846
	50 nM	23,298,426	848,628	723,182	601,483
	500 nM	23,876,136	676,928	567,820	469,150

Supplementary Table 3 | Read counts for RNA-Seq.

	Tg	After filtering and trimming	After non-coding RNA removal	After mapping to genome
Control	0 nM #1	34,273,868	32,193,118	31,631,641
	0 nM #2	28,500,650	26,062,111	25,485,630
	50 nM	33,989,096	25,245,349	24,337,804
	500 nM	28,875,768	23,564,203	22,246,107
NSs	0 nM #1	24,293,960	17,633,378	17,151,384
	0 nM #2	24,572,639	17,823,578	17,464,632
	50 nM	26,623,748	20,144,843	19,700,225
	500 nM	34,822,169	26,913,860	26,374,018
NSs-c1+c2-Ala-mut	0 nM #1	33,055,217	18,538,125	18,135,920
	0 nM #2	32,689,158	21,020,146	20,377,888
	50 nM	35,149,196	20,310,006	19,674,769
	500 nM	29,096,377	16,290,150	15,888,177

Supplementary Table 4 | List of plasmids, related to Figures as indicated.

Plasmid name	Description	Figures
pET28-3C-2B1	For bacterial expression of eIF2Bα, from ref. 4	Figs. 1–3, Supplementary Figs. 1–3
pET28-3C-2B1-A47E	For bacterial expression of eIF2BαA47E, from ref. 4	Supplementary Fig. 2h, i
pETDuet-2B4-2B2	For bacterial expression of eIF2Bδ and β, from ref. 4	Figs. 1–3, Supplementary Figs. 1–3
pCOLADuet-2B5-2B3	For bacterial expression of eIF2Bε and γ, from ref. 4	Figs. 1–3, Supplementary Figs. 1–3
pET28a-d-SFSVNSs	For bacterial expression of SFSV NSs	Figs. 1–3, Supplementary Figs. 1–3
pET28a-d-SFSVNSs-c1-Ala-mut	For bacterial expression of SFSV NSs-c1-Ala-mut (Y5A, Y7A, F33A)	Fig. 2a, d
pET28a-d-SFSVNSs-c2-Ala-mut	For bacterial expression of SFSV NSs-c2-Ala-mut (Y79A, F80A)	Fig. 2a, d
pET28a-d-SFSVNSs-c1+2-Ala-mut	For bacterial expression of SFSV NSs-c1+2-Ala-mut (Y5A, Y7A, F33A, Y79A, F80A)	Fig. 2a, d
pET28a-d-SFSVNSs-TH-Ala-mut	For bacterial expression of SFSV NSs-THAA (T35A, H36A)	Supplementary Fig. 2c
pEBMulti-Neo-human-eIF2alpha-PA	For mammalian expression of eIF2α, from ref. 14	Fig. 2c, d, Supplementary Fig. 2a, h, i
pEBMulti-Neo-human-eIF2alpha-S52A-PA	For mammalian expression of eIF2α-S51A, from ref. 14	Fig. 2c, d, Supplementary Fig. 2i
pEBMulti-Neo-human-eIF2beta	For mammalian expression of eIF2β, from ref. 4	Fig. 2c, d, Fig. 3, Supplementary Figs. 2a, h, i and 3
pEBMulti-Neo-human-eIF2gamma-FLAG-His8	For mammalian expression of eIF2γ, from ref. 4	Fig. 2c, d, Fig. 3, Supplementary Figs. 2a, h, i and 3
pEBMulti-Neo-human-CDC123	For assembly of eIF2 in mammalian cells, from ref. 4.	Fig. 2c, d, Fig. 3, Supplementary Figs. 2a, h, i and 3
pEBMulti-Neo-human-eIF2alpha	For mammalian expression of eIF2α, from ref. 4	Fig. 3, Supplementary Fig. 3

pI.18-3xFLAG	For mammalian expression of 3× FLAG peptide	Figs. 4-6,Supplementary Figs. 4-7
pI.18-SFSVNSs-3xFLAG	For mammalian expression of SFSV NSs protein, from ref. 11	Figs. 4-6,Supplementary Figs. 5-7
pI.18-SFSVNSs-c1+2-Ala-mut-3xFLAG	For mammalian expression of SFSV NSs-c1+2-Ala-mut	Figs. 4-6,Supplementary Figs. 5-7
pCAG-EGFP	For expression of EGFP in rat primary hippocampal neurons & iPS-derived motor neurons	Figs. 5, 6
U6 empty plasmid	For adjusting total amount of DNA for transfection.	Fig. 5
