# Supplementary material for: eIF2B-capturing viral protein NSs suppresses the integrated stress response
Source: Nat Commun. 2021 Dec 7;12:7102. doi: 10.1038/s41467-021-27337-x (PMC8651795; doi:10.1038/s41467-021-27337-x)
Supplement: Supplementary file 2 — Reporting Summary [file 41467_2021_27337_MOESM2_ESM.pdf]

nature portfolio

Corresponding author(s): Yoshiho Ikeuchi
Shintaro Iwasaki
Takuhiro Ito

Last updated by author(s): Nov 12, 2021

Reporting Summary

Nature Portfolio wishes to improve the reproducibility of the work that we publish. This form provides structure for consistency and transparency in reporting. For further information on Nature Portfolio policies, see our Editorial Policies and the Editorial Policy Checklist.

Statistics

For all statistical analyses, confirm that the following items are present in the figure legend, table legend, main text, or Methods section.

n/a	Confirmed	
☐	☒	The exact sample size (*n*) for each experimental group/condition, given as a discrete number and unit of measurement
☐	☒	A statement on whether measurements were taken from distinct samples or whether the same sample was measured repeatedly
☐	☒	The statistical test(s) used AND whether they are one- or two-sided<br>*Only common tests should be described solely by name; describe more complex techniques in the Methods section.*
☒	☐	A description of all covariates tested
☐	☒	A description of any assumptions or corrections, such as tests of normality and adjustment for multiple comparisons
☐	☒	A full description of the statistical parameters including central tendency (e.g. means) or other basic estimates (e.g. regression coefficient) AND variation (e.g. standard deviation) or associated estimates of uncertainty (e.g. confidence intervals)
☐	☒	For null hypothesis testing, the test statistic (e.g. *F*, *t*, *r*) with confidence intervals, effect sizes, degrees of freedom and *P* value noted<br>*Give P values as exact values whenever suitable.*
☒	☐	For Bayesian analysis, information on the choice of priors and Markov chain Monte Carlo settings
☒	☐	For hierarchical and complex designs, identification of the appropriate level for tests and full reporting of outcomes
☐	☒	Estimates of effect sizes (e.g. Cohen's *d*, Pearson's *r*), indicating how they were calculated

Our web collection on statistics for biologists contains articles on many of the points above.

Software and code

Policy information about availability of computer code

Data collection	Automated data collection on the Titan Krios was performed using EPU 2.9.<br>Ribosome profiling and RNA-seq data were collected using Illumina Casava 1.8 software, 3' adapter trimming with Fastp 0.21.0, library sorting based on sample barcode sequence, and random barcode trimming with custom script. rRNA and other non-coding RNA alignment were performed with STAR v2.7.0a. Reads were aligned with STAR v2.7.0a and quantified using custom scripts. Gene annotation was obtained from UCSC Genome Browser (https://genome.ucsc.edu/index.html). In the characterization of neurons, the images acquired with an Axio Observer inverted microscope with ZEN blue and a Nikon confocal microscope (A1R) with NIS elements.
Data analysis	The following softwares used in cryo-EM data processing, model building, and structure validation: RELION-3.1, CTFFIND-4.1, UCSF Chimera-1.15, PHENIX 1.19.2, Coot-0.9.4.1, Buccaneer version 1.6.9 in CCP-EM v1.5.0, and MolProbity 4.5.1.<br>Dissociation constants were calculated with MO.Affinity Analysis v2.2.7.<br>All RNA sequence data were analyzed by DESeq2 (v1.32.0) with custom scripts using R software (v4.0.0) in the RStudio interface (v1.3.1093).<br>Source codes of softwares for ribosome profiling data analysis were at GitHub (https://github.com/ingolia-lab/RiboSeq). GO analysis was performed using iPAGE (v1.2a). ATF4 and GADD34 intensity quantification and sholl analysis were performed with Fiji (http://imagej.nih.gov/ij/, v1.53) and sholl analysis plugin (https://imagej.net/Sholl_Analysis.html). The codes for deep sequencing data analysis have been deposited in Zenodo (DOI: 10.5281/zenodo.5675739).<br>Other custom scripts used in this study will be available from corresponding authors upon request.

For manuscripts utilizing custom algorithms or software that are central to the research but not yet described in published literature, software must be made available to editors and reviewers. We strongly encourage code deposition in a community repository (e.g. GitHub). See the Nature Portfolio guidelines for submitting code & software for further information.

Data

Policy information about availability of data

All manuscripts must include a data availability statement. This statement should provide the following information, where applicable:

- Accession codes, unique identifiers, or web links for publicly available datasets
- A description of any restrictions on data availability
- For clinical datasets or third party data, please ensure that the statement adheres to our policy

The cryo-EM maps and the coordinates of the refined models have been deposited in the Electron Microscopy Data Bank (EMDB) and the Protein Data Bank (PDB) under the following accession numbers: eIF2B•SFSV NSs (EMDB: EMD-32023, PDB: 7VLK), eIF2B•SFSV NSs•1-eIF2 (EMDB: EMD-31474, PDB: 7F66), eIF2B•SFSV NSs•2-eIF2 (EMDB: EMD-31475, PDB: 7F67).
RNA-Seq and ribosome profiling data (GEO: GSE174764) used in this study have been deposited to NCBI.
Other data are provided as a Source Data file. Image J macro script for sholl analysis are provided from the corresponding authors upon appropriate request.

Field-specific reporting

Please select the one below that is the best fit for your research. If you are not sure, read the appropriate sections before making your selection.

☒ Life sciences ☐ Behavioural & social sciences ☐ Ecological, evolutionary & environmental sciences

For a reference copy of the document with all sections, see nature.com/documents/nr-reporting-summary-flat.pdf

Life sciences study design

All studies must disclose on these points even when the disclosure is negative.

Sample size	For RNA-seq and ribosome profiling analyses, two independent biological replicates or single replicate for samples without or with thapsigargin treatment, respectively, were analyzed based on standard in the field, which is sufficient for statistical analysis by DEseq2 to estimate data's dispersion, and also due to cost constraints. No explicit power analyses were used to determine sample size in the other experiments.
Data exclusions	We did not exclude any data for analysis.
Replication	Reproducibility of experiments were confirmed; three independent experiments for microscale thermophoresis, three to five for GDP exchange assay, two or single replicates for samples without or with thapsigargin treatment in deep sequencing, respectively, and three independent experiments for the others. OP-puromycin-labeling and Western blotting data presented in this study are representative results from three independent experiments. For rat neuron culture, five independent experiments for sholl analysis , three independent experiment for the quantification of ATF4 intensity. For iPS cell-derived motor neuron culture, five independent experiments for sholl analysis , three independent experiment for the quantification of ATF4 intensity, five independent experiments for the quantification of GADD34 intensity, three independent experiment for RT-PCR, at least four independent experiment for the quantification of ATF4 intensity with Tg longer treatment.
Randomization	To calculate the gold-standard FSC curves during 3D refinement, the cryo-EM data was randomly split into two halves and refined individually in RELION. For ATF4, GADD34 intensity quantification and Sholl analysis,the target neurons which express GFP used for quantification were randomly selected, however the intensity calculation and measuring the length of neurites were analyzed by automated script and macro equally with no sub-sampling and thus, there was no requirement for randomization.
Blinding	Blinding is not applicable in this study because almost all data were acquired by machines or by custom scripts.

Reporting for specific materials, systems and methods

We require information from authors about some types of materials, experimental systems and methods used in many studies. Here, indicate whether each material, system or method listed is relevant to your study. If you are not sure if a list item applies to your research, read the appropriate section before selecting a response.

Materials & experimental systems

n/a	Involved in the study
☐	☒ Antibodies
☐	☒ Eukaryotic cell lines
☒	☐ Palaeontology and archaeology
☐	☒ Animals and other organisms
☒	☐ Human research participants
☒	☐ Clinical data
☒	☐ Dual use research of concern

Methods

n/a	Involved in the study
☒	☐ ChIP-seq
☒	☐ Flow cytometry
☒	☐ MRI-based neuroimaging

Antibodies

Antibodies used

Anti-FLAG M2 (Sigma-Aldrich, F3165, Lot#SLBG5673V, 1:1000), anti-LC3B (abcam, ab48394, Lot#GR3374546-2, 1:1000), anti-β-actin mAb 6D1 (Medical & Biological Laboratories [MBL], M177-3, Lot#008, 1:1000), anti-β-actin pAb (MBL, PM053, Lot#007, 1:1000), anti-ATF4 rabbit antibody (Cell Signaling Technologies, D4B8, Lot#5, 1:500), anti-GFP mouse antibody (DHSB,DSHB-GFP-1D2,1:50), anti-beta 3 Tubulin mouse antibody (BioLegend, TUJ1, Lot#B249869, 1:1,000), anti-GADD34 rabbit antibody (Proteintech, 10449-1-AP,Lot#00096917, 1:500), IRDye 800CW anti-rabbit IgG (LI-COR, 926-32211, Lot#C91030-13, 1:10000), IRDye 680RD anti-mouse IgG (LI-COR, 925-68070, Lot#C61205-03, 1:10000), IRDye 680RD anti-rabbit IgG (LI-COR, 926-68071, Lot#D00115-05, 1:10000), Alexa Fluor 555-conjugated anti-rabbit IgG (Thermo Fisher Scientific, A27039, Lot#VI311611, 1:1,000) and Alexa Fluor 647-conjugated anti-mouse IgG (Thermo Fisher Scientific, A21235, Lot#VB302731, 1:1,000).

Validation

Anti-FLAG M2, anti-LC3B, anti-β-actin mAb 6D1, and anti-β-actin pAb were validated by the manufacturers based on the molecular weight in Western blot. Anti-ATF4 (ICC staining), anti-GFP (ICC staining), anti-beta 3 Tubulin (ICC staining), anti-GADD34 (ICC staining), IRDye 800CW anti-rabbit IgG, IRDye 680RD anti-mouse IgG, and IRDye 680RD anti-rabbit IgG were validated by the manufacturers based on the detection of proteins in Western blot. Alexa Fluor 555-conjugated anti-rabbit IgG (ICC staining) and Alexa Fluor 647-conjugated anti-mouse IgG (ICC staining) were validated by the manufacturers based on the detection of proteins in immunohistochemistry.

Eukaryotic cell lines

Policy information about cell lines

Cell line source(s)

FreeStyle 293-F Cell Line (Thermo Fisher Scientific, R79007), Flp-In T-REx 293 Cell Line (Thermo Fisher Scientific, R78007), Human iPS cell (Riken cell bank, HPS0076:409B2)

Authentication

Cells were authenticated by periodic morphology check via microscope.
The human iPS cells were handled in accordance with approved protocols by Institute of Industrial Science, The University of Tokyo.

Mycoplasma contamination

All cell lines tested negative for mycoplasma.

Commonly misidentified lines
(See ICLAC register)

No misidentified cell line was used.

Animals and other organisms

Policy information about studies involving animals; ARRIVE guidelines recommended for reporting animal research

Laboratory animals

E18 Slc:Wistar rat, female. Hippocampus from Wistar rat were dissected and hippocampal neurons were dissociated with 0.25% trypsin and DNase.

Wild animals

No wild animals were used in the study.

Field-collected samples

No field collected samples were used in the study.

Ethics oversight

Animal experiment was approved by Animal Experiment Committee of Institute of Industrial Science, The University of Tokyo.

Note that full information on the approval of the study protocol must also be provided in the manuscript.
